# The Mechanism of Metal-Containing Formate Dehydrogenases Revisited: The Formation of Bicarbonate as Product Intermediate Provides Evidence for an Oxygen Atom Transfer Mechanism

**DOI:** 10.3390/molecules28041537

**Published:** 2023-02-05

**Authors:** Hemant Kumar, Maryam Khosraneh, Siva S. M. Bandaru, Carola Schulzke, Silke Leimkühler

**Affiliations:** 1 Institute of Biochemistry and Biology, Department of Molecular Enzymology, University of Potsdam, Karl-Liebknecht Strasse 24 H25, 14476 Potsdam, Germany; 2 Institute for Biochemistry, University of Greifswald, Felix-Hausdorff-Straße 4, 17489 Greifswald, Germany

**Keywords:** molybdoenzyme, formate dehydrogenase, oxygen atom transfer, *Rhodobactor capsulatus*

## Abstract

Mo/W-containing formate dehydrogenases (FDH) catalyzed the reversible oxidation of formate to carbon dioxide at their molybdenum or tungsten active sites. While in the reaction of formate oxidation, the product is CO_2_, which exits the active site via a hydrophobic channel; bicarbonate is formed as the first intermediate during the reaction at the active site. Other than what has been previously reported, bicarbonate is formed after an oxygen atom transfer reaction, transferring the oxygen from water to formate and a subsequent proton-coupled electron transfer or hydride transfer reaction involving the sulfido ligand as acceptor.

## 1. Introduction

The biological reduction of carbon dioxide (CO_2_) involves the conversion of a highly stable and chemically inert compound into more reactive and useful organic chemicals, representing a key process in the global carbon cycle that is of high relevance for combating the greenhouse effect [1]. Among these enzymes, formate dehydrogenases (FDHs) represent a diverse group of enzymes in bacteria, archaea, and eukaryotes that have been proposed to facilitate the reversible two electron and one proton abstraction of formate to produce CO_2_ by catalyzing the following redox reaction, with the equilibrium being on the side of formate oxidation [2,3]:

(1) $${\mathrm{CO}}_{2}+2e^{-}+H^{+}⇌\;{\mathrm{HCOO}}^{-},\;E{}^{\circ}\prime =-420\mathrm{mV}$$

Enzymatic reduction in CO_2_ to formate would allow for the storage of hydrogen as a fuel for industrial applications [4,5,6] as well as carbon sequestration from the atmosphere [1,7], rendering these enzymes interesting targets for biotechnological utilization.

In general, most of the FDH enzymes catalyze preferentially the forward reaction of formate oxidation [8]. The released electrons are transferred in intramolecular electron transfer reactions for the eventual reduction of one of several terminal electron acceptors. Some metal-containing enzymes, however, were described to act rather as CO_2_ reductases [9], reducing CO_2_ to formate [10]. Metal-containing FDHs belong to the family of molybdenum/tungsten cofactor-containing enzymes, binding the bis-Metal-binding pterin Guanine Dinucleotide (bis-MGD) cofactor at their active site [11]. In the bis-MGD cofactor, the metal center is coordinated by two pterin ene-dithiolates, a sulfur atom, and either a cysteine or selenocysteine [12]. FDHs containing the latter ligand were found to be rather oxygen-sensitive [3,12]. All FDHs contain two additional highly conserved residues in the active site, a histidine and an arginine [13,14]. The reaction of formate/CO_2_ interconversion occurs at the molybdenum or tungsten metal ion in the bis-MGD cofactor, in which the metal (M) cycles between the M^VI^, M^V,^ and M^IV^ oxidation states during the reaction [15]. Close to the (bis-MGD) cofactor, a proximal [4Fe–4S] cluster is present in all metal-containing FDH enzymes [16], which is involved in the intramolecular electron transfer reaction. In *Rhodobacter capsulatus* FDH, the oxidation of formate is energetically coupled to the reduction in oxidized nicotinamide adenine dinucleotide (NAD^+^) in the cytosol [17].

While metal-dependent FDH enzymes have been studied for several decades and the *E. coli,* the FdhF enzyme was among the first molybdoenzymes to be crystalized [12]. Details of the reaction mechanism remain only poorly understood and the overall catalytic mechanism of formate oxidation is still under debate [2,18].

It was reported [19,20,21] that the reaction of metal-containing FDHs do not constitute the characteristic oxygen atom transfer reaction, as typically catalyzed by the DMSO reductase family of Mo/W bis-MGD-containing enzymes [22]. Instead, it has been suggested that the reaction involves only the heterolytic fission of the C-H bond facilitated through a direct hydride transfer to the terminal sulfido ligand at the molybdenum atom, with the immediate product being CO_2_ and not bicarbonate [19,20]. This hypothesis was first proposed by Thauer et al. (1975) in the characterization of the FDH (ferredoxin: CO_2_ oxidoreductase) from *Clostridium pasteurianum* [19]. It was later supported by a study from Khangulov et al. (1998) [21] on *Escherichia coli* FdhF, who investigated product formation using GC-MS with ^13^C-labeled formate in ^18^O-enriched water, which resulted in the detection of only ^13^CO_2_ gas without any ^18^O atoms.

A recent study by Meneghello et al. (2021) [20] applied an electrochemical approach to the tungsten-containing FDH from *Desulfovibrio vulgaris* Hildenborough [23] to confirm conclusions from earlier cumulative work; i.e., that the substrate of FDHs for the back reaction of CO_2_ reduction is CO_2_ rather than HCO_3_^–^. However, as already pointed out by Cooper et al. (1968) [24], it still remains possible that these observations are more a reflection of the binding of the preferred molecule to the active site and do not necessarily reflect what is happening during substrate turnover, especially since the product formation was not quantified in the assay, and by electrochemistry it cannot be determined which substrate is bound at the active site and which ligands are involved in the binding. It, therefore, might be possible that charged HCO_3_^−^ is hindered from entering the active site via the hydrophobic channel that has been identified in crystal and cryo-EM structures of FDH enzymes, while CO_2_ in contrast can easily enter [23,25]. At the active site, CO_2_ could react with H_2_O to form HCO_3_^−^ which then acts as the actual substrate for the reaction, which is only formally the reduction of CO_2_ [19]. This option has, so far, been unconsidered in most respective studies [2,10,13,15,18,21,23]. It should, however, attract more attention, since in some X-ray structures of FDH enzymes a water molecule was identified in the active site which is bound in the vicinity to the conserved arginine residue in the second coordination sphere [23,26,27]. It is not unlikely that H_2_^18^O progression into the active site of the enzyme and its exchange with unlabeled water therein is slow, if the enzyme and active site are saturated with unlabeled water. This might be one of the reasons why Khangulov et al. did not detect an ^18^O-labeled CO_2_ product in their approach [21].

Here, we reinvestigated the oxygen atom transfer from H_2_O^18^ to formate yielding C^18^O^16^O and present the identification of bicarbonate as the first intermediate in the reaction of formate oxidation, before CO_2_ is produced in the secondary reaction. We provide clear evidence that metal-containing FDHs catalyze a typical oxygen atom transfer (OAT) reaction. This work revises the 25-year-old hypothesis that FDHs represent an exception in the family of Mo/W-containing enzymes by catalyzing a heterolytic C-H bond cleavage reaction instead of OAT [21]. We also show for the back reaction, that CO_2_ and not bicarbonate enters the active site, likely by the proposed hydrophobic channel, but at the active site, first bicarbonate is formed likely in a carbonic anhydrase-like reaction before formate is released, as evidenced by using the enzyme soaked in H_2_^18^O labeled water and a ^13^CO_2_ saturated buffer, which resulted in the production of H^13^C^16^O^18^O^−^ labeled formate.

## 2. Results

### 2.1. ^13^C-Labeled Formate Oxidation by RcFDH in the Presence of ^18^O-Labeled Water

**Figure 1:**
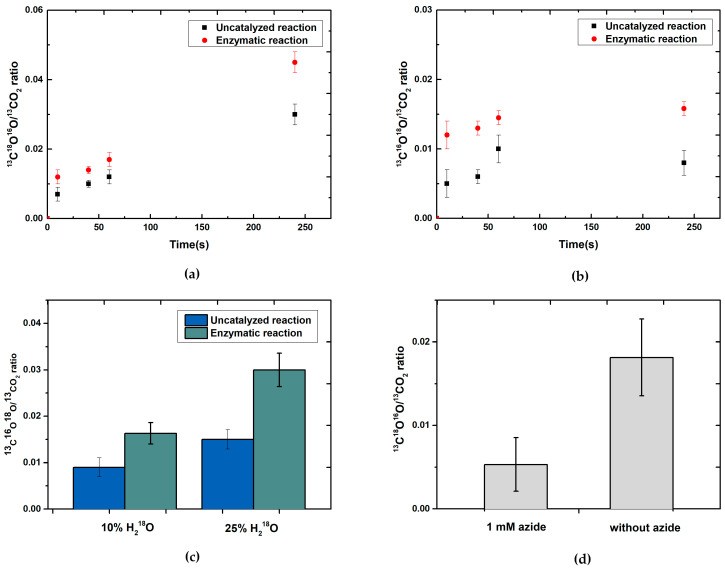
Time-dependent ratios of CO_2_ isotopomer (^13^C^18^O_2_/^13^CO_2_) formation at 10 °C (**a**) and 25 °C (**b**) using *R. capsulatus* FDH (pH 9) to monitor the enzymatic oxidation of ^13^C-labeled formate in the presence of 10% H_2_^18^O. The reaction was started by the addition of 30 µM of FDH preincubated with H_2_^18^O to MS vials which contained 10 mM H^13^COO^–^ 5mM NAD^+^ in 100 mM Tris-HCl, pH 9. To avoid any changes in equilibrium, samples were taken directly at the indicated timepoints. Headspace samples were analyzed by GC-MS. The uncatalyzed reaction contained the same components without the enzyme. Additionally, up to 100 mM of NaH^13^CO_3_ was used in uncatalyzed reactions as a ^13^CO_2_ source to monitor the secondary reaction of non-enzymatic CO_2_ hydration. (**c**) Ratios of CO_2_ isotopomer (^13^C^18^O_2_/^13^CO_2_) formation after 40 s using different amounts of H_2_^18^O for ^13^C-labeled formate oxidation using *R. capsulatus* FDH at pH 9 (25 °C). (**d**) ^13^C labeled formate oxidation using *Rc*FDH in the presence of 1 mM sodium azide and 10% H_2_^18^O. The reaction was started by the addition of 5 µM FDH preincubated with H_2_^18^O to an MS vial which contained 10 mM H^13^COO^−^_,_ 5 mM NAD^+^ in 100 mM Tris-HCl, pH 9. The headspace sample was taken after 10 s and analyzed by GC-MS. The sample with azide contained an additional 1 mM of sodium azide.

We reanalyzed the experiment performed in 1998 by Khangulov et al. [21] with a few modifications using purified *R. capsulatus* FDH, ^13^C-labeled formate, and ^18^O-enriched water to determine the amount of ^18^O in the immediate ^13^CO_2_ reaction product. The usage of *R. capsulatus* FDH is advantageous compared to *E. coli* FDH since the enzyme is more oxygen stable, whereby the experiment can be performed in the absence of high inhibitor concentrations of, e.g., azide [28]. Azide might interfere with the reaction as it is a mixed-type inhibitor to formate and interacts with both the CO_2_ and the formate-binding moieties in the active site [29,30]. Further, the reaction of formate oxidation of *R. capsulatus* FDH is 77 times slower as compared to *E. coli* FdhF, which facilitates detecting the reaction product [17,31]. However, *R. capsulatus* FDH has a pH optimum of 9.0, which is expected to increase the equilibrium constant of the secondary ^13^CO_2_ + H_2_^18^O⇆H_2_^13^COO^18^O⇆H^13^COO^18^O^−^ + H^+^ reaction [17]. To avoid interferences with the secondary reaction at higher pH, we performed the reaction at 10 °C and 25 °C in comparison (Figure 1a,b) since at temperatures below 20 °C and in the absence of carbonic anhydrase, the non-enzymatic hydration of CO_2_ is slow [24].

Mass spectrometric analysis of the isotopic composition of CO_2_ gas, released upon enzymatic oxidation of ^13^C-labeled formate (^13^COO^−^) in ^18^O enriched water (H_2_^18^O), was performed to study the formation of ^18^O enriched CO_2_ (^13^C^16^O^18^O) [21]. When H_2_^18^O is used as the oxygen atom source, H^13^C^16^O_2_^18^O^–^ bicarbonate is the direct formate oxidation product from which ^13^C^16^O^18^O is produced (as a major product along with minor amounts of C^16^O_2_) in the secondary reaction by the dehydration of bicarbonate according to the following overall reaction sequence:

H^13^COO^−^ + H_2_^18^O⇆H^13^C^16^O_2_^18^O^−^ + 2 H^+^ + 2e^−^ and H^13^C^16^O_2_^18^O^−^ + H^+^⇆^13^C^16^O^18^O + H_2_^16^O (2)

^13^C-labeled formate oxidation by *Rc*FDH in the presence of 10% ^18^O labeled water was carried out at pH 9 and the ratio of the product isotope content for [^13^C^16^O^18^O]/[^13^C^16^O^16^O] was determined after 10 s, 40 s, 1 min, and 4 min (Figure 1a) at 10 °C and 25 °C (Figure 1a,b).

In the uncatalyzed reaction, NaH^13^CO_3_ was also converted to ^13^C^16^O^18^O in the chemical equilibration of bicarbonate, carbonic acid, and CO_2_ but substantially more slowly. We obtained a ratio of 0.005 after 10 s for the uncatalyzed reaction and a ratio of 0.012 for the catalyzed reaction at 10 °C (Figure 1a). At 25 °C, the ratio for the uncatalyzed reaction increased to 0.006 after 10 s and to 0.03 after 4 min (Figure 1b).

The catalyzed reaction, however, produces higher relative amounts of ^13^C^16^O^18^O compared to the uncatalyzed reaction under any investigated circumstances, and these ratios are significantly higher in the cold throughout, as well as after 4 min at 25 °C. This supports oxygen from water being primarily inserted into CO_2_ enzymatically and not only by an uncatalyzed secondary hydration of ^13^CO_2_ with H_2_^18^O from the solvent. After 10 s, the ratio of 0.012 in the catalyzed reaction is lower than the theoretically expected value of 0.066, which most likely goes back to a slow H_2_O/H_2_^18^O exchange rate in the enzyme active site. After 40 s, 1 min, and 4 min, the ratio increased to 0.016 in the enzymatic reaction (Figure 1a). When the percentage of labeled H_2_^18^O content was raised from 10 to 25%, a two-times higher ratio [^13^C^16^O^18^O]/[^13^C^16^O^16^O] was observed after 40 s at 10 °C (Figure 1c), demonstrating that H_2_^18^O exchange in the enzyme is a limiting factor at this temperature. The higher ratios of the catalyzed reaction differentiate it from the background reaction and show that FDH catalyzed the oxygen atom transfer from H_2_^18^O to ^13^C-labeled formate yielding ^13^C^16^O^18^O.

In contrast to the report by Khangulov et al. [21], we determine an immediate insertion of ^18^O from H_2_^18^O water into CO_2_ already 10 s after the start of the reaction, underlining that an oxygen atom transfer reaction for this enzyme has to be considered. The different observations then and now might be based on the absence of the inhibitor azide in our study, while in the previous investigation of the *E. coli* enzyme, 3 mM azide was present to slow down the reaction. When the reaction was performed with only 1 mM azide added and the *R. capsulatus* enzyme, indeed, almost no ^18^O labeled ^13^CO_2_ was detected within the first 10 s of the reaction (Figure 1d).

### 2.2. Solvent Deuterium Kinetic Isotope Effect on the Formate Oxidation Reaction

The results above indicate that FDH catalyzes an oxygen atom transfer reaction, in which water (or hydroxide) might displace the formal hydride leaving the group in formate, a mechanism that has been observed also in enzymes like phosphite dehydrogenase [29]. Consequently, we also determined the solvent kinetic isotope effect (KIE) using D_2_O and H_2_^18^O water in the reaction. Previous kinetic isotope effect studies using deuterium-labeled formate demonstrated significant isotope effects on V_max_ and V_max_/*K*_m_^formate^ at lower formate concentrations, indicating that cleaving the C–H bond is at least partially rate-determining for the enzymatic reaction [15,30]. Solvent kinetic isotope studies with D_2_O or H_2_^18^O have not been performed before on FDH enzymes. D_2_O has only been used in EPR studies on FDH enzymes from *Cuprividus necator* and *Desulfovibrio desulfuricans* [15,32] showing that in the sample prepared in D_2_O, the proton coupling to the Mo^V^ signal disappeared based on the much weaker nuclear magnetic moment of ^2^H relative to ^1^H, which demonstrates the solvent exchangeability of the coupled proton. The solvent-exchangeable proton was conclusively interpreted to be bound to the sulfido ligand of the molybdenum center, while the non-solvent exchangeable proton was interpreted to be bound to the Cα atom of the amino acid ligand (Cys in case of *Rc*FDH).

**Figure 2:**
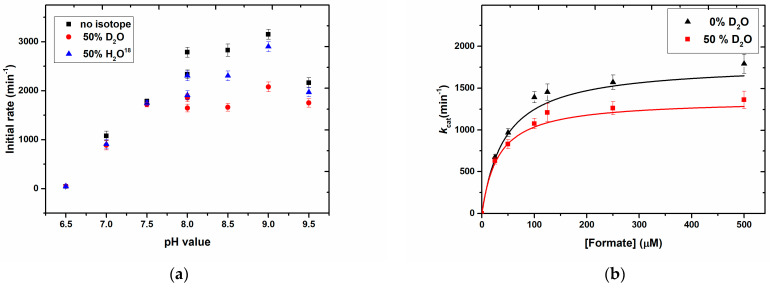
(**a**) Kinetic solvent isotope effect at different pH using 50% D_2_O and 50% H_2_^18^O. The shown ratios were obtained at different pH values. Phosphate buffer (100 mM) for pH 6.5–8.0 and Tris-HCl buffers (100 mM) for pH 8.0–9.5 were used. (**b**). Kinetic solvent isotope effect on steady-state kinetics using H_2_O with 0% and 50% D_2_O. Reaction mixture contained 2 mM NAD^+^ and formate (50–500 µM) in phosphate buffer (100 mM, pH 7.9). The measurement at 340 nm was started after adding *Rc*FDH (50 nM). pH and pD values were corrected, as per the literature [7,33].

We determined the pH-dependent solvent kinetic isotope effect (KIE) on the rapid reaction kinetics using D_2_O and H_2_^18^O. The primary D_2_O KIE ratios for the reaction, as listed in Table 1, exhibit a decrease at higher pH values (Figure 2a). This implies that the deprotonation of water is one of the rate-limiting steps and the KIE is less pronounced at higher pH, when water is more easily deprotonated (p*K*_a_ 14). The pre-steady state KIE with D_2_O, which was also used in combination with DCOO^−^ formate, shows significant isotope effects on V_max_ at lower D_2_O concentrations (Figure 2b), an effect which was not/not substantially increased when HCOO^–^ was used as the substrate (Table 2). This confirms that water, and hence the oxygen atom transfer reaction, has an effect on the reaction rate in addition to the C-H bond cleavage (or C-D bond cleavage in DCOO^−^).

### 2.3. Steady State Kinetics with Monothioformate

We synthesized a sulfur-containing analog of formate, monothioformate, in which one of the oxygen atoms is replaced by sulfur to obtain more insight into the reaction intermediate.

**Figure 3:**
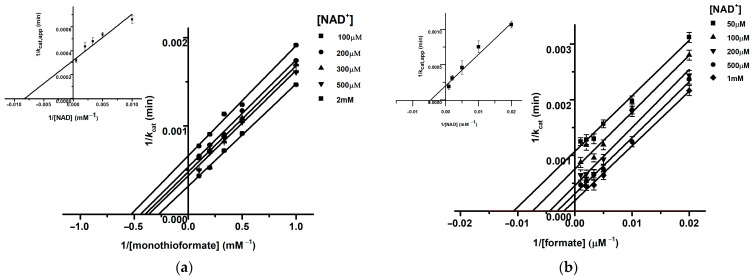
(**a**). Bisubstrate kinetics of the monothioformate oxidation reaction with NAD^+^ as electron acceptor. Lineweaver–Burk plot of k_cat_ using 7 nM FDH with varying thioformate concentrations of 0.5–20 mM and constant NAD^+^ concentrations of 100 µM, 200 µM, 300 µM, 500 µM, and 1 mM measured in 100 mM Tris-HCl, pH 9, at room temperature). Inset: the *K*_i_ value was obtained from the secondary plot of the apparent 1/V_max_ of the Linewaever–Burk plot. The data represent the mean values of three independent measurements (±standard deviation). (**b**) Bisubstrate kinetics of the formate oxidation reaction with NAD^+^ as electron acceptor. Lineweaver–Burk plot of k_cat_ using 7 nM FDH with varying formate concentrations of 0.5–20 mM and constant NAD^+^ concentrations of 500 µM, 100 µM, 200 µM, 500 µM, and 1 mM measured in 100 mM Tris-HCl, pH 9, at room temperature. Inset: the *K*_m_ and k_cat_ values were obtained from the secondary plot of the apparent 1/*k*_cat app_ intercepts of the Lineweaver–Burk plot. The data represent the mean values of three independent measurements (±standard deviation).

The kinetic constants of purified *R. capsulatus* FDH were determined following the reduction in NAD^+^ as the terminal electron acceptor using synthesized monothioformate and dithioformate as substrates. The *K*_m_ values for NAD^+^ and monothioformate were calculated to be 111.5 ± 24 µM and 3.3 ± 0.24 mM, respectively, with a *k*_cat_ of 3032 ± 83 min^−1^ from the secondary plot (Figure 3a and Table 3). Taking into account that only 44% of the purified protein was catalytically active in this experiment, the *k*_cat_ can be calculated as 6890.9 min^−1^ for a fully functional enzyme. The double reciprocal plots with varying monothioformate and NAD^+^ concentrations revealed a ping-pong mechanism for the bisubstrate reaction of the enzyme (Figure 3a). Since we have previously proposed a ternary complex mechanism with formate as a substrate, we reinvestigated formate oxidation and also obtained a ping-pong mechanism with a *K*_m_^formate^ of 0.145 ± 0.025 mM, a *K*_m_^NAD+^ of 159.12 ± 12 mM and a *k*_cat_ for the fully active enzyme of 7500 min^−1^ (Table 3). We must, therefore, revise our previously published conclusions and report here that *R. capsulatus* FDH, such as the FDHs from *C. necator* [15] and *E. coli* FdhF [34], also catalyzes formate oxidation and NAD^+^ reduction according to a ping-pong mechanism (Figure 3b). The distinct results obtained before are based on too high enzyme concentrations that were used in the respective assay [17]. Most notably, it is shown here that monothioformate acts as a potent substrate for FDH, with *k*_cat_ values comparable to formate, but with an observed increase in *K*_m_ of a factor of 22 (Table 3).

**Figure 4:**
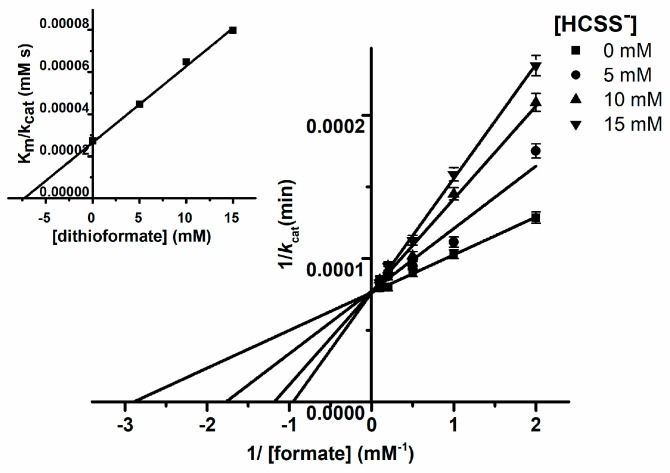
Inhibition kinetics of *Rc*FDH with dithioformate. Steady state kinetics were carried out under anaerobic conditions using 2 mM benzyl viologen (ε_578, benzyl viologen_ = 8.65 mM^−1^cm^−1^) as an electron acceptor. The assay mixture contained different concentrations of formate (0.05–5 mM), dithioformate (0–15 mM) and 2 mM benzyl viologen in 100 mM Tris-HCl buffer, pH 9. Photometric measurement at 578 nm was carried out for 60 s after the addition of 30 nM *Rc*FDH.

In contrast, dithioformate was not accepted as a substrate by *R. capsulatus* FDH and acted instead as a competitive inhibitor (Figure 4), with a *K*_i_ of 7.5 mM calculated from the secondary plot.

### 2.4. Nuclear Magnetic Resonance Experiments for the Identification of the Reaction Intermediate

Nuclear magnetic resonance (NMR) spectroscopy was chosen to verify the results reported above by a method that not only confirms the elemental composition of the analytes but also their chemical structure and relative quantity. The observed resonance shifts are directly correlated to the chemical environment of the investigated nucleus. They can be calculated relatively reliably prior to measurement using incremental contributions of binding partners toward the final resonance as a sum of all induced upfield and downfield shifts. However, in the case of this study, all resonances of relevance could be validated based on the literature available. With NMR spectroscopy, it is possible to quantify the relative abundance of the species in solution, which bear NMR-sensitive nuclei. In the case of formate dehydrogenase, the immediate product (bicarbonate/carbonate), as verified by all prior experiments in the course of this investigation and its decomposition product (CO_2_), do not bear hydrogen atoms or do not bear those hydrogen atoms, which would be suitable for detection by ^1^H-NMR (no rapid exchange allowed). It was, therefore, necessary to turn to less sensitive ^13^C-NMR spectroscopy, which needs more time per measurement to lead to unambiguous data and a good signal-to-noise ratio (only 1.1% relative abundance of the NMR active nucleus). To counteract this disadvantage and in order to be able to monitor substrate conversion to intermediate (bicarbonate) and the final product (CO_2_), it was attempted to slow the enzymatic turnover down by low temperature, pH conditions unfavorable for enzymatic performance, and/or the addition of azide. Still, the enzymatic reactions were too fast to receive unambiguous catalytic data for the transformation by NMR spectroscopy. In order to decrease the duration of the ^13^C-NMR measurements, ^13^C-labeled formate was then used as a substrate. The time for each ^13^C-NMR measurement could, therefore, be reduced to ca. 25 min per run. It was possible to receive a time-dependent series of ^13^C-NMR spectra with a partial azide-inhibited enzyme at a low temperature (5 °C) and a moderately high pH of 9 monitoring the changes in the NMR resonances of ^13^C-labeled formate, bicarbonate, and CO_2_. Formate, according to the literature, exhibits a resonance in the ^13^C-NMR spectrum at 170.5 ppm [35], bicarbonate resonates at 161.3 ppm [36], and CO_2_ at 124.0 ppm [37]. The resonances found in this study are in excellent agreement with these published data with 171.37 ppm, 161.28 ppm, and 125.57 ppm, respectively, considering that distinct conditions were applied (see the spectrum in SI, Figure S1). To be entirely certain of the correct assignment and the absence or presence of carbon-bound hydrogen atoms, in addition to the normally proton-decoupled ^13^C-NMR spectra, proton-coupled spectra were recorded, which confirmed the presence of a C-H moiety for formate and the absence of the same for bicarbonate and CO_2_ signals (Figure S2 in the SI).

**Figure 5:**
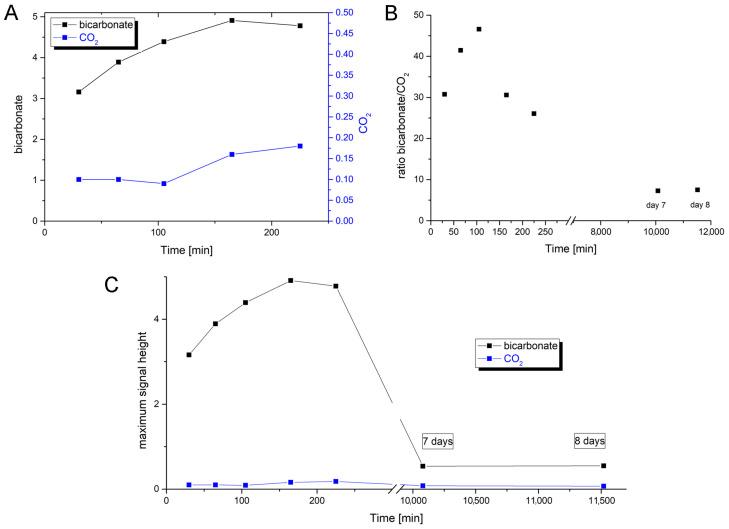
Time-dependent abundance of intermediate and final products of enzymatic formate oxidation. (**A**) The development of the maxima (signal height) of the resonance signals for bicarbonate (black, left y-axis) and CO_2_ (blue, right y-axis) up to 225 min. (**B**) Time-dependent development of the ratio of bicarbonate content versus CO_2_ content derived from the maxima of the resonance signals in the NMR spectra. This includes measurements after 7 and after 8 days to confirm that the bicarbonate/CO_2_ equilibrium has settled. (**C**) The development of maxima (signal height) of the resonance signals for bicarbonate (black) and CO_2_ (blue) with a joint y-axis reflecting their relative abundance in the NMR sample tube over time. This includes measurements after 7 and after 8 days to confirm that the bicarbonate/CO_2_ equilibrium has settled. The experiment was carried out at 5 °C and pH 9 with an azide-inhibited enzyme and a ^13^C-labeled formate substrate.

The results of the time-dependent experiment indicate that the production of bicarbonate precedes the formation of CO_2_, even though a small amount of CO_2_ was already present in the first measurement. The substrate was given in large excess and was not consumed by the end of the series. Figure 5A shows the time-dependent development of the maxima of the resonance signals of bicarbonate and CO_2_. CO_2_ abundance in the NMR sample tube does only increase significantly after the formation rate of bicarbonate already starts to decrease. Unfortunately, the initial steep rise from zero bicarbonate to the first measurement could not be observed directly, since the enzymatic turnover is still too fast for the time that a single ^13^C-NMR measurement requires. CO_2_ formation, in contrast, accelerates, while bicarbonate formation is already slowing down. This firmly points to bicarbonate as the primarily formed species. The bicarbonate/CO_2_ equilibration in an aqueous solution should start immediately upon the formation of either species in an aqueous solution, even at a pH of 9, and both hypothetical enzymatic mechanisms are generally conceivable: (i) initial formation of CO_2_, which together with water forms carbonic acid, that is then quickly deprotonated at this pH; or (ii) initial formation of bicarbonate, which in aqueous solution becomes partially protonated to carbonic acid and then splits into CO_2_ and water. In the former scenario (i) it would be impossible, though, that the ratio of bicarbonate versus CO_2_ first rises and then decreases, since the formation of bicarbonate would be entirely dependent on the presence of CO_2_ in the first place. Figure 5C shows the development of the bicarbonate:CO_2_ ratio over time including measurements after 7 and after 8 days of sample tube storage in the cold, when the concentration of bicarbonate and CO_2_ has apparently reached equilibrium under the applied conditions. This equilibrium has decidedly lower bicarbonate contingent compared to the measurements directly after the enzymatic reaction was started, which is further evidence for the primary formation of bicarbonate and only a secondary, possibly combined enzymatic/non-enzymatic, formation of CO_2_ which follows the enzymatic transformation of formate by oxidation and oxygen atom binding to the carbon center.

In Figure 5C, it can be seen that the overall content of bicarbonate and CO_2_ together is decreasing after having reached a maximum at approximately 165 min. The observation further confirms that after the initial production of bicarbonate, this first enzymatic product is transformed into CO_2_ which then, likely, leaks into the gas phase of the sample tube. The tube’s gas phase does not contribute to the measured signal. From the tube, CO_2_ can further leak slowly into the environment since the chosen tube model was not gas-tight for safety reasons. Following the formation of the enzymatic product bicarbonate and its partial transformation to CO_2_, the solution, for a short period of time, becomes oversaturated in CO_2_ until eventually the equilibrium is reached with regard to both the reversible conversion between CO_2_ and bicarbonate and the solubility of CO_2_.

While the obtained data of ^13^C-labeled formate substrate are unambiguous regarding the order of product occurrence, the concomitance of the primary enzymatic reaction and secondary equilibration was still unfavorable for observing continuous product enrichment. In order to vary the reaction kinetics, ^13^C-labeled thioformate was used as a substrate instead of formate, since it was shown in the bisubstrate kinetics that this compound can be used as a substrate by *R. capulatus* FDH with similar *k*_cat_ values as for formate. These experiments were carried out at room temperature and pH 9 without the addition of an azide inhibitor. The expected signals could be assigned based on the literature values for thioformate (range of 200.5 to 202.9 ppm) [35], thiocarbonate (186.4 ppm) [38], and COS (154.2 ppm) [37]. In this study, the resonances were found in measurements in varied conditions at ca. 211 ppm, 183 ppm, and 159 ppm, respectively; i.e., all slightly shifted from the literature data but unambiguously identified through control and coupling experiments. Notably, (i) the observed resonance for the initial product suggests thiocarbonate rather than thiobicarbonate according to the respective literature and (ii) the final product COS is confirmed to have decidedly better solubility in an aqueous solution than CO_2_ since it is polarized (SciFinder provides the value 35 g/L which was calculated for the database/search engine using Advanced Chemistry Development (ACD/Labs) Software V11.02 (© 2023–2021 ACD/Labs)). COS is clearly the dominant species in the thiocarbonate/COS equilibrium even though monothiocarbonic acid is more acidic than carbonic acid (p*K*_a1_ (H_2_CO_2_S) = 3.2 [39]; p*K*_a1_ (H_2_CO_3_) = 6.35 [40]; N.B.: for carbonic acid, this is the apparent p*K*_a_ which is higher than the actual p*K*_a1_ of 3.88 [41]; the discrepancy goes back to the equilibrium with CO_2_). The measurement duration was set in this series to the regular 60 min to obtain spectra of high quality (little noise), sacrificing the glimpse at a more immediate sample composition right after the start of the enzymatic catalysis.

**Figure 6:**
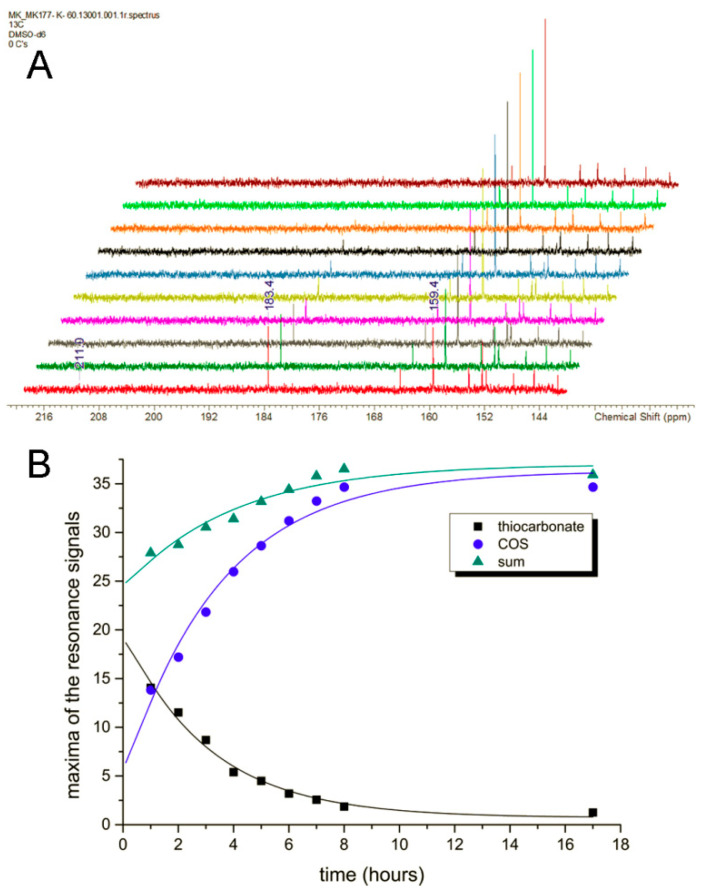
Time-dependent abundance of intermediate and final products of enzymatic thioformate oxidation (**A**) The ^13^C NMR spectra were recorded every hour up to hour 9. The last spectrum (brown) was measured at 17 h. The resonance signal of thiocarbonate at 183.4 ppm constantly decreases, while that of COS at 159.4 ppm constantly rises. Only a small amount of the substrate (monothioformate at 211.0 ppm) is left in the first spectrum (red). (**B**) The development of the resonance signal maxima over time for thiocarbonate (black squares), COS (blue dots), and their sum (dark cyan triangles) shown with their respective asymptotic fits as solid lines. N.B.: the fits are not meant as proper kinetic evaluation but are added in order to approximate and visualize the development before the first collected data points and the sum of the two products. Asymptotic fits y = a – b × c^x^: monothiocarbonate: a = 0.71709 (+/− 0.5452); b = −19.23708 (+/− 1.01177); c = 0.72343 (+/− 0.02679); COS: a = 36.33576 (+/− 1.31434); b = 31.7694 (+/− 2.09314); c = 0.74924 (+/− 0.03287); sum: a = 37.01988 (+/−1.04481); b = 12.77271 (+/−1.41994); c = 0.77727 (+/−0.05322). The experiment was carried out at 25 °C and pH 9 with the enzyme bearing a minor amount of residual azide from protein preparation and with a ^13^C-labeled thioformate substrate.

The spectra recorded over time are shown with a range that includes thiocarbonate and COS plus various signals from the NAD^+^/NADH pair (Figure 6A). In the first spectrum which was recorded (and which is essentially a cumulative summary of ca. 1000 recorded scans throughout this first hour), the concentrations of immediate product thiocarbonate and final product COS are almost identical, with thiocarbonate being only marginally more abundant. However, the abundance of thiocarbonate thereafter decreases regularly while the abundance of COS increases regularly and the total abundance first increases slightly (indicating further enzyme activity) and then reaches a plateau (Figure 6B). This proves that the sample has not yet reached enzymatic and solubility equilibrium prior to completion of the first spectrum’s recording and, more importantly, that the occurrence of thiocarbonate cannot follow the production of COS but the order must be reversed; i.e., COS can only be formed after the enzyme has produced thiocarbonate (otherwise, the concentration of the latter would not be decreasing). The data firmly support the initial formation of thiocarbonate, followed by transformation to COS, with which it is in an equilibrium that has (in contrast to the formate experiments) a high gas-to-anion ratio; i.e., here, the equilibrium lies on the side of COS. Since this ratio throughout the NMR monitoring constantly increases after the complete consumption of a monothioformate substrate, COS cannot be the initial product of the enzymatic transformation.

The applied concentration of the enzyme is lower than the observed abundance of monothioformate, at least in the early spectra. This implies that the primary product monothiocarbonate is able to leave the active site or even the enzyme entirely, at least under the conditions of these NMR experiments.

### 2.5. Bicarbonate Is Formed as a Direct Substrate in the Reaction of CO_2_ Reduction

**Figure 7:**
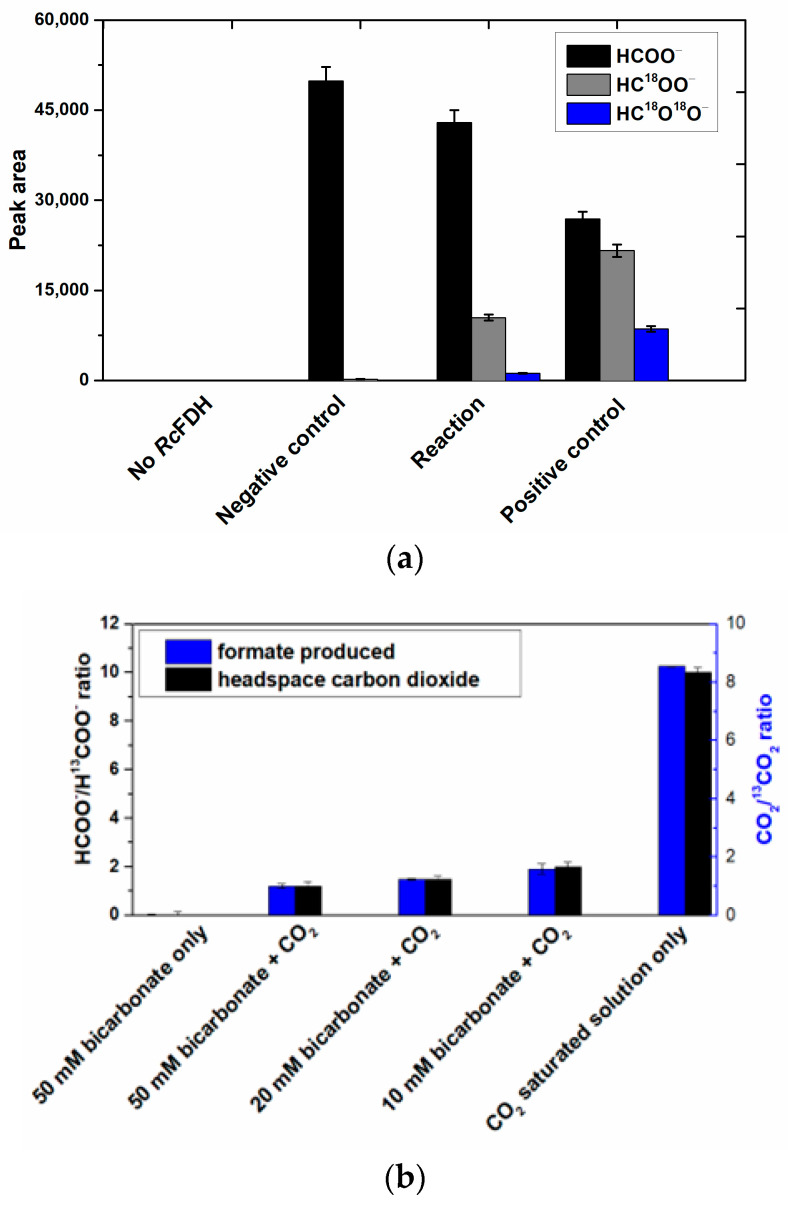

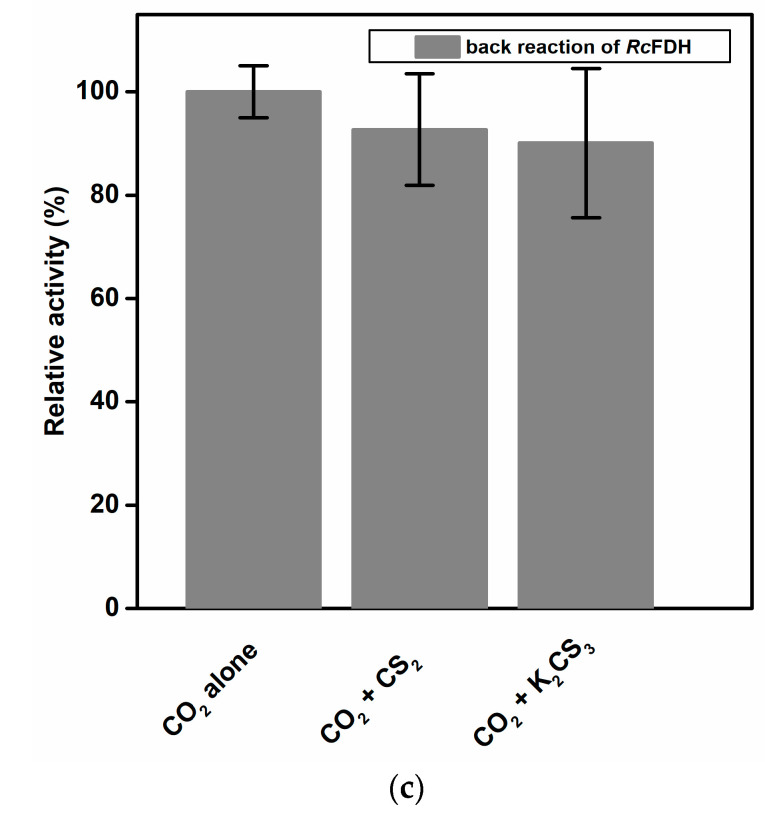
(**a**) CO_2_ reduction reaction of *Rc*FDH in the presence of H_2_^18^O. Reaction mixture of 100 µL contained 2 mM reduced methyl viologen and CO_2_-purged phosphate buffer (100 mM, pH 6.6). The reaction was started by adding the *Rc*FDH (5 µM) and H_2_^18^O (50%) mixture into the solution anaerobically at 25 °C. The negative control had no H_2_^18^O added to it and in the case of the positive control, H_2_^18^O and CO_2_ were mixed 30 min prior to *Rc*FDH addition. Reactions were stopped after 10 s by adding 30 µL of acetone followed by freezing in liquid nitrogen. Samples were derivatized and analyzed by GC − MS, as detailed in the Methods section. (**b**) The CO_2_ reduction reaction of *Rc*FDH using CO_2_ and/or NaHC^13^O_3_ as substrates. The reaction volume of 100 µL contained 2mM reduced methyl viologen and a CO_2_ saturated phosphate buffer (100 mM, pH 6.6) with different concentrations of NaHC^13^O_3_ solution. The reaction was started by adding *Rc*FDH (5µM) anaerobically at 25 °C. Reactions were stopped after 10 s by adding 30 µL of acetone followed by freezing in liquid nitrogen. Samples were derivatized and analyzed by using GC−MS. Black bars represent the ratios of normal and ^13^C-labeled formate produced. Blue bars represent the headspace CO_2_/^13^CO_2_ ratios analyzed by GC − MS before the addition of *Rc*FDH. (**c**) The effect of CS2 and K_2_CS_3_ on the back reaction of *Rc*FDH. A reaction volume of 100 µL contained 1mM NAD^+^ and a CO_2_-saturated phosphate buffer (100 mM, pH 6.6). A total of 150 mM of carbon disulfide (CS_2_) and 5 mM of potassium trithiocarbonate were added to check inhibition. The reaction was monitored at 340 nm for 1 min after adding *Rc*FDH (5 µM) anaerobically at 25 °C.

The results presented above show that for *R. capsulatus* FDH, bicarbonate is the final product of enzymatic formate oxidation that leaves the enzyme as such or is transformed to CO_2_ in a secondary reaction within the enzyme. To determine whether CO_2_ or bicarbonate are the substrates that enter the enzyme for the back reaction of CO_2_ reduction, we investigated the reduction in the CO_2_ substrate in more detail. Assuming that bicarbonate based on its charge cannot enter the oxidized active site through the proposed hydrophobic CO_2_ channel, the formation of bicarbonate directly in the active site appears necessary, e.g., in a carbonic anhydrase-like reaction after CO_2_ binding. Indeed, in some of the crystal structures (*D. vulgaris* Hildenborough [23], formyl-methanofuran dehydrogenase [27]), a water molecule was found attached to the conserved arginine residue close to the molybdenum/tungsten center in the active site. To analyze whether *R. capsulatus* FDH converts CO_2_ to bicarbonate in the active site which is then used as a direct substrate to produce formate, we incubated the enzyme first in H_2_^18^O-labeled water, before a CO_2_ saturated buffer and reduced methyl viologen were added to the mixture. After derivatization of the resultant formate with 2,3,4,5,6-pentafluoro-benzylbromide (Figure S8 in the SI), we analyzed the product with GC-MS for the presence of ^18^O. After 10 s of the enzymatic reaction in 50% H_2_^18^O at 25 °C, the formation of formate-bearing labeled oxygen (HC^18^O^16^O^−^) could indeed be verified (Figure 7a). This confirms that added CO_2_ is hydrated with H_2_^18^O within the enzyme or most likely directly at the active site before its use as a substrate in the reduction to formate. In the absence of either enzyme or H_2_^18^O in the solution, no HC^18^O^16^O^–^ was detected (Figure 7a). In another control reaction, H_2_^18^O was first incubated with a CO_2_-saturated buffer for 30 min to yield C^16^O^18^O in the secondary non-enzymatic hydration of ^12^CO_2_ before FDH was added. In this case, both HC^18^O^16^O^−^ and HC^18^O^18^O^−^ were detected as enzyme products (Figure 7a). Formate standard in the absence of enzyme incubated with H_2_^18^O did not show any formation of HC^18^O^16^O^−^ (data not shown in Figure 7). It can, therefore, be excluded that formate exchanges oxygen atoms with water spontaneously in a non-enzymatic/non-catalyzed reaction.

In order to identify the substrate species for the reduction which enters the active site, we mixed different ratios of labeled NaH^13^CO_3_ (at high pH) and unlabeled CO_2_ purged buffer (at low pH) solutions as substrates and analyzed the formate isotope that was enzymatically formed by *Rc*FDH within the first 10 s of the reaction (Figure 7b). Assuming a negligible non-enzymatic hydration of CO_2_ within the first 10 s, unlabeled formate as a major product indicates that CO_2_ entered the active site much more efficiently than the ^13^C-labeled bicarbonate or even exclusively under the experimental conditions. In contrast, finding ^13^C-labeled formate as a major product would have indicated that bicarbonate entered the active site preferentially. We observed relatively higher amounts of ^12^C formate (Figure 7b), indicating that a substrate entered the enzyme mostly in the ^12^CO_2_ form and not as H^13^CO_3_^−^. We also looked at the headspace CO_2_/C^13^O_2_ ratio of the reaction in a separate experiment to simulate the conditions of the period in time when the enzyme was added. These ratios correlate quite well with the formate product carbon isotope ratios indicating that the gaseous form of a substrate enters the enzyme (Figure 7b).

It is further shown that neither trithiocarbonate nor CS_2_ were used as substrates for the back reaction. Likely, trithiocarbonate, like bicarbonate, cannot enter the active site through the hydrophobic channel. Since CO_2_ is apparently converted to bicarbonate in the active site in a carbonic anhydrase-like reaction, it is unsurprising that CS_2_ cannot be used as a substrate because the hydration of CS_2_ is substantially slower, while it evidently does interfere with the active site and/or the hydrophobic channel and, therefore, inhibits the reduction in CO_2_ when present (Figure 7c).

## 3. Discussion

CO_2_ is a kinetically and thermodynamically stable molecule, with a high negative reduction potential value for the CO_2_/HCOOH pair (highly pH dependent), all of which render its activation and reduction difficult tasks [1]. For the biological reversible conversion of CO_2_ to formate, prokaryotes and eukaryotes use FDH enzymes [2,3]. FDHs are a heterogeneous and broadly distributed group of enzymes that evolved to be part of diverse metabolic pathways, most notably the generation of energy from formate oxidation by coupling it to the reduction in several terminal electron acceptors, or the reduction in CO_2_ into formate catalyzed by some prokaryotic organisms. FDHs belong to two major classes, the metal-dependent and the metal-independent ones [9]. The metal-dependent ones are only present in prokaryotes and were shown to catalyze the oxidation of formate with higher catalytic efficiencies as compared to the metal-independent enzymes [13]. In particular, metal-dependent enzymes are much better catalysts for the reduction in CO_2_, and for a long time it was believed that metal-independent enzymes are not even able to catalyze the back reaction [10]. Metal-dependent enzymes belong to the class of Mo- or W-containing enzymes bearing the bis-MGD cofactor [11]. This class of Mo- and W-containing enzymes was first shown by Holm and coworkers in the 1980s to catalyze classical oxygen atom transfer (OAT) reactions [42]. In OATs, the oxygen atom from water is transferred to the substrate which is oxidized, or in the opposite direction, from the substrate to yield water; these reactions are coupled to the reversible transfer of two electrons and two protons in the course of the transformation cycles [22]. The electrons are directly transferred to the Mo/W metal ion of the cofactor and the metals cycle between the Mo/W^IV^ and Mo/W^VI^ oxidation states, with Mo/W^V^ as the intermediate state. FDH enzymes were considered to be an exception in the group of molybdenum- and tungsten-containing enzymes for catalyzing direct hydride abstraction from the parent carbon atom instead of an oxygen atom transfer [2,13,18,21]. Such exceptional behavior was mainly proposed based on a report by Khangulov et al. in 1998 [21], concluding that the immediate product of formate oxidation is CO_2_ and not bicarbonate. They used an experimental setup with ^18^O-labeled water and ^13^C-labeled formate and observed only ^13^C^16^O_2_ as an initial product of the reaction. This experiment and its outcome have never really been questioned since and resulted in the proposal of numerous mechanisms for FDH-catalyzed formate oxidation without considering any OAT transitions [2,10]. However, a uniformly accepted, undisputed reaction mechanism has not been put forward as of yet. Since the enzyme in the Khangulov et al. experiment was inhibited by relatively high amounts of azide [21], which was used to slow down the reaction, we decided to reinvestigate the experiment in the absence of azide. We used *R. capsulatus* FDH instead of *E. coli* FDH, an enzyme that we characterized in detail before and which is much more oxygen-tolerant compared to the *E. coli* FdhF enzyme [17]. This enabled us to use low/no azide concentrations with the enzyme for the experiments. We performed the formate oxidation assay at 10 °C to particularly slow down the secondary reaction of non-enzymatic CO_2_ hydration [24]. Our data clearly show that after a reaction time of 10 s, labeled ^13^C^18^O^16^O was readily detectable, demonstrating that the oxygen of H_2_^18^O water is, in fact, inserted into the product. The enzyme-catalyzed reaction was much faster as compared to the secondary hydration of CO_2_ under our experimental conditions, as evidenced by respective control reactions.

We further show solvent kinetic isotope effects on the reactions using D_2_O, H_2_^18^O, and DCOO^–^, confirming the impact of water on the substrate transformation rates and, therefore, a mechanistic role of H_2_O. It has been shown previously that the D of DCOO^–^ of formate is transferred to the sulfido ligand on the Mo-centre [15,30,32], with which our results are entirely in accordance. To accurately determine the rate-limiting step of the reaction, more detailed investigations will be necessary during future studies. In our experimental setup, we worked with a 50% H_2_^18^O saturated buffer which reliably gave rise to the observed solvent kinetic isotope effect.

**Figure 8:**
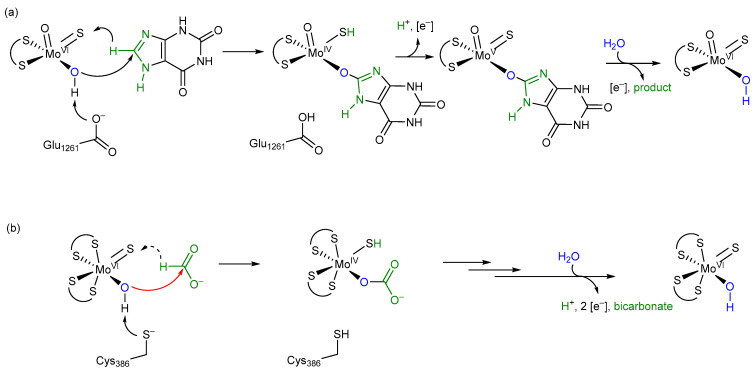
(**a**) The mechanism for xanthine oxidase adapted from Ref. [22]. (**b**) A tentatively proposed short mechanism for FDH operating via oxygen atom transfer. The similarity of the two substrates at the site of transformation is emphasized in green. The arrow shown in red constitutes the attack of the active site on the substrate which initiates OAT, which is immediately followed by a hydride or proton-coupled electron transfer to the sulfido ligand. In both mechanisms, the product is released (bicarbonate in the case of FDH) and water is bound to the active site.

To further confirm the insertion of oxygen into H^13^COO^–^-labeled formate and to determine the formed product intermediate, we used NMR as a detection method. Since the reaction was too fast and the ^13^C-NMR measurements were relatively time-intense, we had to slow down the reaction with azide. The reaction was further performed at 5 °C to also decelerate the non-enzymatic hydration of CO_2_. The first product that was detected by NMR in substantial abundance was bicarbonate, the formation of which already began to decrease before the abundance of CO_2_ increased up to its maximum reaching even an oversaturation of the solution. In a previous report, the same observations were made, detecting bicarbonate as the first intermediate by NMR [43]. The first data point, however, was drawn only after 25 min, which impedes disentangling the still quite fast enzymatic reaction and the subsequent secondary reaction of the CO_2_/HCO_3_^–^ equilibrium at pH 9.0. Therefore, in order to receive even more coherent data, we used ^13^C-labeled thioformate in the experiment, which was shown to be a suitable substrate of *R. capsulatus* FDH in the bisubstrate kinetic experiments. When ^13^C-labeled thioformate was used in the NMR experiment with low azide concentrations (remnants of protein preparation), thiocarbonate was clearly detected as the first intermediate, the abundance of which then decreased while COS abundance increased steadily. Here, thiocarbonate is detected instead of thiobicarbonate, based on the fact that thiobicarbonate is more acidic than bicarbonate, less stable at this pH, and easily deprotonated. This clearly confirms the oxygen atom transfer with bicarbonate/thiocarbonate as reaction intermediates of the reaction, before CO_2_ or COS are formed in a secondary follow-up reaction inside the enzyme and/or a non-enzymatic secondary reaction outside the enzyme. We further investigated the back reaction of CO_2_ reduction to clarify whether bicarbonate enters the enzyme (preferentially) or whether CO_2_ does instead, followed by bicarbonate formation at the active site. Using a ^12^CO_2_ saturated buffer in the presence of a H_2_^18^O saturated enzyme, we obtained H^12^C^18^O^16^O^−^labeled formate in the first 10 s of the reaction, showing that CO_2_ is the primary substrate that enters the enzyme, which is then converted to a H^12^C^18^O^16^O_2_ bicarbonate at the active site (possibly in a carboanhydrase-like hydration reaction). This bicarbonate is then used as the actual substrate for the reduction to formate and water. When H^13^CO_3_^−^ was used as the only substrate, no ^18^O-labeled formate was detected. Likely, bicarbonate is not used as a direct substrate in the back reaction because it cannot enter the enzyme through the hydrophobic CO_2_ channel. Or, the entrance is hindered by the histidine, since structural changes were observed in oxidized and reduced X-ray structures [26]. In the NMR experiment, using ^13^C-labeled formate, bicarbonate was detected as an immediate intermediate product and much higher in abundance than the enzyme, suggesting it may be released from the enzyme. In this reaction, an azide-inhibited enzyme was used. For carbonic anhydrase, it was reported that azide is a competitive inhibitor for bicarbonate dehydration [44], while it is a non-competitive inhibitor for CO_2_ hydration. For *E. coli* FdhF, inhibition studies with azide showed that azide acts as a competitive inhibitor for formate oxidation and as a non-competitive inhibitor for the reduction in CO_2_ [30], with the inhibitor being more competent toward the oxidation of formate. For *Rc*FDH, it was recently shown that azide acts as a mixed-type (competitive, non-competitive) inhibitor for both formate and CO_2_ [45,46]. It was concluded in the study of the *E. coli* enzyme that the inhibitors bind differently to the reduced and oxidized forms of the enzyme. Assuming a similar inhibition mechanism for azide with bicarbonate/CO_2_ in *R. capsulatus* FDH, we propose that in the reaction of formate oxidation, the bicarbonate binding site is competitively blocked by azide so that the dehydration of bicarbonate is inhibited and bicarbonate is released instead of CO_2_, likely through the formate channel. The formate channel might be suitable for more charged substrates, such as formate, nitrate, and bicarbonate, while the CO_2_ channel is specific for CO_2_ and cannot be used by bicarbonate [25]. Redox, charge, and protonation states of the active site should all have an impact on the attraction or repulsion of charged and uncharged substrates/products. Therefore, the presence/absence of substrates, products, inhibitors, reducing, and/or oxidizing agents might have gatekeeper function(s) for either of the substrates. Further, after reduction in the enzyme with formate, conformational changes in the second coordination sphere were observed in the crystal structures of the *D. vulgaris* FDH enzyme [23] and the reinterpreted structure of the *E. coli* FdhF enzyme [47]. We, therefore, hypothesize that by the structural rearrangement in the formate reduced enzyme, the formate funnel becomes accessible for the release of bicarbonate, an exit site that is blocked by the histidine in the oxidized enzyme or inhibited enzyme. We assume that the site for CO_2_ hydration and bicarbonate dehydration is close to or at the conserved arginine residue in the second coordination sphere, since in the crystal structures of the *D. vulgaris* [23] and formyl-methanofuran dehydrogenase from *Methanothermobacter wolfeii* [27], a water molecule was identified to reside in the vicinity of this residue. In previous studies, it was also speculated that azide is bound to this residue in the active site, matching our hypothesis that this is the non-competitive binding site for azide and bicarbonate [32,45]. Overall, we want to propose the oxygen atom transfer mechanism for the reversible oxidation of formate by metal-containing FDH enzymes, as shown in Figure 8, which shows similarities to well-accepted enzyme mechanisms, such as the one for xanthine oxidase. We think that all metal-containing FDHs would work after this mechanism and that there are no differences in W- or SeCys-containing enzymes

Considering the difference between formate and xanthine at the site of enzymatic transformation being only the presence of two N functionalities versus two oxygen functionalities and a relatively similar bonding situation thereof, the oxygen atom transfer mechanism of FDH is tentatively proposed to resemble that of XO (xanthine oxidase), with the –OH groups of the initial species stemming from water [22] (Figure 8). All protonation or deprotonation states of substrates, amino acids, and ligands will be directly dependent on the concentration of protons in the active site, i.e., its pH value. For a more detailed mechanistic insight into the actual individual transformative steps, a number of further experiments will need to be carried out.

The release of the bicarbonate product, one proton, and two electrons from the inner active site composition regenerates the species with which this cycle starts. The key step of this proposed mechanism constitutes a typical and common oxygen atom transfer, as implied by our presented new data and as proposed for xanthine oxidase. This mechanism is in accordance with all undisputed experimental data available, stoichiometrically balanced and, hence, coherent.

In our mechanism, we propose that after formate binding the amino acid ligand at the Mo atom is displaced by water, which might be facilitated by a conformational change in the enzyme upon formate binding. The displacement of the cysteine ligand in *R. capsulatus* FDH has been shown by us in previous studies by iodoacetamide labeling of the nitrate-inhibited and formate-reduced enzyme and by EXAFS studies [13,14]. The iodoacetamide labeling of the selenocysteine ligand was also shown for the *E. coli* enzyme [13]. However, not all enzymes are inhibited by iodoacetamide, e.g., the *D. vulgaris* Hildenborough enzyme [23]. In this enzyme, no carboxamidomethyl labeling of the selenocysteine ligand was observed. Notably, in this experiment, a nitrate and a glycerol-inhibited enzyme were used, and in particular glycerol might interfere with the accessibility of iodoacetamide to this enzyme [23]. Further, selenocysteine-containing enzymes are more oxygen sensitive, and it remains possible that after the displacement of the selenocysteine ligand, the selenocysteine is easily oxidized and the oxidized SeO_2_ or SeO_3_ species do not react with iodoacetamide. We also do not exclude that distinct FDH enzymes have different accessibility for iodoacetamide, e.g., by variations of amino acids in the formate-binding funnel. EXAFS studies of the *R. capsulatus* enzyme further proved displacement of the cysteine residue in the formate-reduced enzyme by an oxygen atom (which can be the one from water as shown in this study). In the EXAFS data of the azide or cyanate-inhibited enzyme, instead of cysteine sulfur a light atom was observed to be bound to the molybdenum center [28], which we assigned to be an oxygen atom from water rather than a C or N atom from the inhibitors which are proposed to not directly bind to molybdenum. The binding of water observed in the EXAFS studies supports our oxygen atom transfer mechanism. In contrast, for the *E. coli* FdhF and *D. vulgaris* enzymes, EXAFS and crystallographic studies did not support displacement of the selenocysteine ligand [23,48]. We consider it likely that the *E. coli* enzyme was oxidatively damaged and the sulfido ligand was lost in the enzyme preparation for the EXAFS studies, while in the formate reduced structure of the *D. vulgaris* enzyme, the oxidation state of the molybdenum atom is not clear and the crystalized enzyme might be in the re-oxidized Mo^VI^ state after product release since neither the product nor the substrate was present in the structure. Often, EPR studies are considered as an argument for the oxidation state-dependent active site structure of the enzyme. However, the Mo^V^ active site constitutes an intermediate state after product release and one electron oxidation in which the amino acid ligand is quite likely to rebind again to the molybdenum atom which otherwise would be coordinatively unsaturated [18,49]. In previous studies, several groups proposed a hydride transfer mechanism with the formate being bound within the second coordination sphere of the active site metal [15,50]. One of the arguments used by the authors to support their mechanism was that the second p*K*_a_ value of formic acid (i.e., the one for C-H dissociation) disfavors a proton abstraction and the resulting carbanion would be unstable. In our mechanism, the formate is directly bound to the molybdenum atom through an oxido function derived from a water molecule, so a carbanion would not be formed after proton abstraction [2]. Nevertheless, in our mechanism, we leave it open whether the hydrogen of formate is transferred as a hydride or in a proton-coupled electron transfer reaction to the sulfido ligand, forming the SH group and the reduced Mo^IV^ (i.e., we do not propose the direction in which the electrons or electron pairs move when entering the transition state). The sulfido group as a hydride (or proton) acceptor has been well-established in XDH enzymes [51] and was proven in FDH enzymes by EPR studies to contain a strongly coupled, solvent exchangeable and substrate-derived proton with a hyperfine constant of 20–30 MHz, which is consistent with the hydrogen atom from the formate being transferred to this ligand in the first coordination sphere of the molybdenum upon reduction. This observation is also consistent with our model reaction mechanism; however, it does not prove that the H-atom is transferred as a hydride.

Meneghello et al. (2021) [20] recently chose an electrochemical approach with the tungsten-containing FDH from *Desulfovibrio vulgaris* Hildenborough for confirming the substrate of FDHs for the back reaction of CO_2_ reduction being CO_2_ rather than HCO_3_^−^, as previously and repeatedly concluded before. However, we want to point out that product formation was not measured in this study and that in their experimental setup, the enzymes might have been washed away; therefore, their results need to be taken with caution. As already emphasized by Cooper et al. (1968) [24] and confirmed by our investigation, respective studies do not necessarily reflect what is happening directly at the active site. We, therefore, propose that for the reduction of CO_2_ the intermediate ionic species HCO_3_^−^ is hindered from entering the reduced active site via the hydrophobic CO_2_ channel that has been identified in crystal and cryo-EM structures, while CO_2_ instead can easily enter. This is still consistent with the report by Meneghello et al. (2021) [20]. At the active site, CO_2_ then reacts with H_2_O to form HCO_3_^−^ which subsequently provides the direct substrate for the back reaction resulting in formate and water formation through oxygen atom transfer from bicarbonate. In conclusion, we also want to emphasize that mechanistic studies of metal-containing FDH enzymes need to be performed in the absence of any inhibitor under strictly anaerobic conditions.

## 4. Materials and Methods

### 4.1. Chemicals and Reagents

Elemental potassium was purchased from Sigma-Aldrich (St. Louis, MI, USA). ^13^C-labeled formic acid (^13^C, 99%) was purchased from Cambridge Isotope Laboratories, Inc. (Tewksbury, MA, USA). Phenol was purchased from Fisher Scientific (Waltham, MA, USA). POCl_3_ was purchased from Acros (Geel, Belgium). Penta fluorobenzyl bromide (PFBBr) was purchased from Alfa Aesar (Haverhill, MA, USA). Potassium hydrogenphosphate and potassium dihydrogenphosphate were purchased from Sigma-Aldrich. Potassium dithioformate was prepared according to a literature method [52].

### 4.2. Synthesis of Monothioformate

First, the starting materials KHS and phenyl formate had to be synthesized.

(i) Synthesis of potassium hydrogen sulfide (KHS). Potassium hydrogen sulfide, according to literature reports, is most commonly synthesized by the reaction between a solution of potassium sulfide with excess dihydrogen sulfide. However, the such-synthesized potassium hydrogen sulfide is not completely pure. To avoid water and any other impurities, the following modified procedure was used to synthesize dry and pure potassium hydrogen sulfide. A total of 12 mL of dry ethanol was charged in a pre-evacuated flask which was equipped with a stirrer and bubbler under nitrogen. The flask was cooled to −78 °C (a mixture of isopropanol and liquid nitrogen was used as a cooling bath). Then, 1.7 g of potassium was inserted into the flask gradually and cautiously in small portions over a period of 2 h. When all of the potassium particles had dissolved, dry gaseous dihydrogen sulfide which was produced through a Kipps apparatus and dried over CaCl_2_, which was passed through the reaction mixture for about 4 h. As soon as the passing of dihydrogen sulfide started, the formation of a white precipitate could be observed. The passing of dihydrogen sulfide was continued until the end of precipitation. Then, the excess gaseous dihydrogen sulfide was removed by bubbling nitrogen through the solution. The solid compound was dried under a vacuum for 3 h. Elemental analysis (calcd). S 44.43, H 1.397; found S 43.11, H 1.80; IR (KBr): ν cm^–1^ = 2520 (w), 2100 (br), 1650 (s), 1400 (br), 1250 (w), 1150 (m), 1000 (s), and 700 (w).

(ii) Phenyl formate was synthesized with small modifications to a literature procedure [53]. A total of 1.2 eq of ^13^C-labeled formic acid (21.2 mmol, 0.975 g) was mixed with 1 eq of phenol (17.67 mmol, 1.66 g) in a three-neck round bottom flask under a nitrogen atmosphere. The reaction mixture was stirred for 3 h at 80 °C under nitrogen. Then, the reaction mixture was cooled to room temperature and 0.33 eq of POCl_3_ (5.83 mmol, 0.89 g) was added slowly and dropwise. The reaction mixture was poured slowly into a solution of sodium bicarbonate in ice water. The resultant solution was then extracted three times with diethyl ether (40 mL). The combined organic phases were dried over sodium sulfate, filtered, and the solvent was removed from the filtrate with a rotary evaporator at a low temperature to obtain crude phenyl formate. Phenyl formate is not stable and should be used immediately for the following step. ^1^HNMR (CDCl_3_, 400MHz, 298 K): δ: 8.37 ppm (1H, CH), δ: 7.43 ppm (2H, CH-Ar), δ: 7.26 ppm (1H, CH-Ar), δ: 7.07 ppm (2H, CH-Ar); ^13^CNMR (CDCl_3_, 400MHz, 298 K): δ: 161.12 ppm (1C, CH), δ: 155.54 ppm (1C, C), δ: 115.75 ppm (2C, CH), δ: 130 ppm (2C, CH), and δ: 121.10 ppm (C, CH).

(iii) Potassium monothioformate was synthesized with small modifications to a literature procedure. A total of 1 eq of phenyl formate (18 mmol, 2.20 g) was mixed with 1 eq of dry KHS (18 mmol, 1.30 g) in a Schlenk flask under nitrogen. The Schlenk flask was placed into an ultrasonic bath at 0 °C for 2 h. Then, 2 mL of dry methanol was added to the Schlenk flask and the mixture was stirred for another 2 h. After the addition of 20 mL of dry diethyl ether, the reaction mixture started to form a precipitate. After precipitation stopped, anaerobic filtration was carried out under a nitrogen atmosphere. The precipitate was washed with small amounts of cold methanol and dried under a vacuum to yield a fine pale yellow powder. ^1^HNMR (CDCl_3_, 400MHz, 298 K): δ: 9.8 ppm and δ: 10.4 ppm (1H, CH) and ^13^CNMR (CDCl_3_, 400MHz, 298 K): δ: 211.11 ppm (1C, CH). Two resonances were detected due to the tautomerization effect. MS (ESI): *m/z* = 101.4 [M] H^13^COSK. IR (KBr): ν_max_ = 2580 (C-H stretching), 1630 (C=O stretching), 1390 (C-H bending), 812 (C-S). Elemental analysis (calcd). C 11.99, H 1.01, and S 32.00; found C 11.96 and H 1.54; S 31.41.

### 4.3. NMR Spectroscopy

NMR spectra were recorded on a Bruker Avance II 300 spectrometer (300, 75, and 121.5 MHz, respectively). Chemical shifts (δ) are given in parts per million (ppm) using solvent signals as a reference (DMSO-d6 ^1^H: δ = 2.50 ppm; ^13^C: δ = 39.52 ppm; CD_3_OD ^1^H: δ = 3.31 ppm; ^13^C: δ = 49.15 ppm) relative to external tetramethylsilane (δ = 0 ppm).

### 4.4. Preparation of the Time-Dependent NMR Measurement Sample for the Enzymatic Reaction of RcFDH with ^13^C-Labeled Sodium Formate

In order to prepare the nicotinamidadenindinucleotide stock (150 mM), 99.5 mg of NAD^+^ was dissolved in 1 mL of a tris(hydroxymethyl)aminomethane buffer (75 mM, pH: 9). For preparing the sodium azide stock (50 mM), 3.25 mg of sodium azide was dissolved in 1 mL of a buffer. After the preparation of the stocks, 290 µL of NAD^+^ stock was charged in a new vial. Then, 100 µL of labeled sodium formate (1.62 M; 11.2 mg in buffer at pH 9), 24 µL of sodium azide, and 150 µL of a buffer were added, respectively. Finally, and immediately before the NMR measurement series started, 60 µL of an enzyme (360 µM; for preparation see below) was inserted into the reaction mixture. A total of 0.5 mL of the prepared mixture was transferred quickly to the NMR tube. Deuterated methanol (CD_3_OD) was used as the internal standard in an insert tube (i.e., not mixed with the sample solution). The ^13^C time-dependent measurements were started immediately without filtration of the sample at 5 °C and run in automation overnight.

### 4.5. Preparation of the Time-Dependent NMR Measurement Sample for the Enzymatic Reaction of RcFDH with a 13C-Labeled Monothioformate

NAD^+^ stock solution was prepared as described above. A total of 290 µL of NAD^+^ stock was charged in a new vial. Then, 100 µL of monothioformate (1.12 M; 11.2 mg in buffer at pH 9) and 150 µL of water were added. Finally, 60 µL of an enzyme (360 µM) was added to the sample mixture immediately before the measurements started. A total of 0.5 mL of the prepared mixture was transferred quickly to the NMR tube. Deuterated DMSO-d6 (dimethylsulfoxide) was used as the internal standard in an insert tube (i.e., not mixed with the sample solution). The ^13^C time-dependent measurements were started immediately without filtration of the sample solution at room temperature (25 °C operation temperature in the NMR laboratory) allowing for continuous monitoring of the transformations.

### 4.6. Control Measurements for the Thioformate Substrate Series

The same procedure as described above was followed with regard to sample preparation, except that one component or more were left out (Table 4). The total volume of the reaction mixture was 600 µL in all cases, of which 5 mL was transferred to the NMR tube. The solution volume with the ingredient not included in the control mixture was replaced by the buffer to reach the total volume of 600 µL.

In none of the control condition experiments could any progress of the enzymatic reaction be observed. The resonances at 183.4 ppm for thiocarbonate and 159.4 ppm for COS remained absent. Although NAD^+^ has many ^13^C-NMR signals in a broad range of the spectrum, these do not interfere with the signals of the enzymatic transformation of interest. In contrast to all other components of the reaction, FDH enzyme concentration is so low that no respective signals could be observed. Tris(hydroxymethyl)aminomethane (THAM) was used for the preparation of the buffer (pH = 9), which has two resonances in the ^13^C-NMR spectrum (56.14 ppm and 61.92 ppm). An additional coupling ^13^C-measurement (4 h duration) for the reaction mixture bearing all components proved that the intermediate at 183.4 ppm is not showing any splitting in the ^13^C {H} coupled NMR. This means that there is no proton in scalar coupling with this carbon atom, which further supports an assignment to the thiocarbonate dianion (Figure S5).

### 4.7. The Expression and Purification of R. capsulatus FDH

*R. capsulatus* FDH was expressed in E.coli MC1061 cells containing plasmids pTHfds05 and pTHfds07 obtained from a previous study [17] and purified according to the published procedure [25]. The enzyme was stored in a 75 mM Kpi buffer containing 10 mM sodium azide, pH 7.5, and before usage of the enzyme in the assays, the buffer was exchanged with a 75 mM Kpi buffer, pH 7.5, using PD-10 desalting columns (Sephadex G-25 M; Amersham Biosciences) to remove azide.

### 4.8. ^13^C-Labeled Formate Oxidation by RcFDH in the Presence of ^18^O-Labeled Water

The oxidation of ^13^C-labeled formate by *Rc*FDH in the presence of ^18^O-labeled water was carried out to analyze the resultant CO_2_ isotope composition. The reaction mixture of 100 µL was prepared in a glass insert containing a GC vial by mixing 10 mM ^13^C-labeled formate and 5 mM NAD^+^ anaerobically in 100 mM Tris-HCl, pH 9. *R. capsulatus* FDH, pre-mixed with ^18^O-labeled water, was anaerobically transferred to a syringe. The final concentration of the enzyme and ^18^O-containing water were 10–30 µM and 10–50%, respectively. The reaction was started by injecting the assay mixture into the closed GC vial at 10 °C. For control reactions, up to 100 mM NaH^13^CO_3_ was used as a ^13^CO_2_ source. All the reagents were pre-incubated at the required temperatures beforehand. Headspace samples were taken from the vial by the autosampler after the indicated time intervals. The isotopic composition of produced ^13^CO_2_ was analyzed by using GC-MS QP2010 SE (Schimadzu) modified for headspace samples. Sample volumes of 1–5 µL were used in the DB-WAX UI column (30 m × 0.32 mm × 0.25 µm, Agilent). The injection temperature was kept at 200 °C. The temperature program for analysis consisted of (i) 30 °C for 3 min; (ii) from 30 °C to 200 °C at 30 °C/min; (iii) 200 °C for 1 min; and (iv) from 200 °C to 30 °C at 30 °C/min.

For MS analysis, the selected ion monitoring (SIM) mode was used due to its high sensitivity. A detector voltage of +0.3 kV relative to tuning with the ion source and interface temperature of 200 °C was applied each. The MS analysis method was used to look for *m*/*z* values corresponding to the ^13^C^16^O^16^O, ^13^C^16^O^18^O, and C^13^O^18^O^18^ isotopomers of CO_2_. Different concentrations of NaH^13^CO_3_ solutions were used to confirm the correct *m*/*z* values and to create a calibration curve (Figure S9).

### 4.9. Derivatization and Analysis of the Formate Using GC-MS

The formate was derivatized by modifying a previous method [54] using 2,3,4,5,6-pentafluoro-benzylbromide(PFBBr). A total of 100 µL of enzyme-free samples were mixed with 50 µL of a 325 mM phosphate buffer, pH 8.5. To this, 365 µL of 100 mM PFBBr solution prepared in acetone was added. The solution was vortexed for 1 min and heated at 60 °C for 20 min. After cooling down to room temperature, 500 µL of *n*-hexane was added and vortexed for 1 min. Phases were separated by centrifugation at 13,000 rpm for 1 min and the upper organic phase was carefully pipetted into a 2 mL insert containing GC vials. The samples were analyzed by using GC-MS QP2010 SE (Schimadzu) modified for headspace samples. Sample volumes of 1 µL were used in the DB-WAX UI column (30 m × 0.32 mm × 0.25 µm, Agilent).

### 4.10. The Solvent Kinetic Isotope Effect Using Pre-Steady State Kinetics

The solvent kinetic isotope effect for the reductive half-reaction was studied using deuterated water (D_2_O) in comparison to H_2_O. An SX-20 stopped-flow spectrophotometer (Applied Photophysics, Inc., Leatherhead, UK) was used to study the reaction of the formate using purified R. capsulatus FDH. A total of 5 µM of FDH was anaerobically equilibrated in a 100 mM Kpi buffer, pH/pD = 7.9, to remove the azide present from the purification. pD of the deuterated buffer was adjusted 0.4 units higher in the pH meter (pD = pH + 0.4). Glucose oxidase (10 IU) and catalase (100 IU) were mixed with enzymes to remove the residual oxygen from the stopped-flow cell. The preparation was filled into a 10 mL glass syringe anaerobically. The second syringe was filled with the anaerobically prepared formate or deuterated formate solution (5 mM) in the same buffer and was containing 5 mM glucose. Since the stopped-flow instrument was not in an anaerobic chamber, additional modifications from Applied Photophysics, Inc. were adopted. Additional nitrogen was continuously purged around the syringe housings of the instrument. The water bath was purged with nitrogen for 30 min to remove oxygen. Both photomultiplier tube (PMT) and photodiode array (PDA) detectors were used alternatively depending on the type of experiment. The path length used for the experiments, unless mentioned otherwise, was 1 cm. The reaction of the formate with as-isolated *R. capsulatus* FDH was performed as described by Niks et al. (2016) [15] at 10 °C. The formate was used at saturating concentrations and enzyme concentrations used were between 2.5–15 µM, depending on the experiment. In the case of the PMT detector, changes at 450 nm were followed. In the case of the PDA detector, spectra were recorded between 280–700 nm wavelengths. The curves obtained at 450 nm were fitted to the sum of three exponentials using non-linear least square regression analysis using the following equation:

$$A_{t}=A_{\infty }\pm \sum A_{n}\;\exp\left(-\frac{t}{k_{n}}\right)$$

where *n* represents the number of kinetic phases. Data analysis was performed using ProData Viewer 4.2.0 (Applied Photophysics, Inc.).

### 4.11. The Solvent Kinetic Isotope Effect at Different pH Values

The effect of solvent isotopes (D_2_O and H_2_^18^O) at different pH was determined anaerobically by following the formation of NADH at 340 nm. A 100 mM phosphate buffer was used at pH values between 6.0 and 7.5 and a 100 mM Tris HCl buffer was used at pH values between 8.0 and 9.0. A D_2_O/H_2_O or H_2_^18^O/H_2_O ratio of 0.5 was chosen for all the measurements by adding 50% D_2_O, H_2_^18^O, or H_2_O to the assay mixture. The reaction volume of 100 µL contained 10 mM of formate, 2 mM NAD^+^, and the indicated buffer. The reaction was started by adding 25 nM FDH to the cuvette, followed by photometric detection at 340 nm for 60 s.

### 4.12. Steady State Kinetics

*Rc*FDH kinetic assays were measured anaerobically using a UV-2401PC spectrophotometer (Shimadzu Europa, Duisburg, Germany) and following the formation of NADH at 340 nm (ε_NADH_ = 6220 m^−1^·cm^−1^). Steady state kinetics for monothioformate were determined by varying the concentrations of monothioformate (0.5–20mM) and NAD (0.05–2mM). In the case of formate, the concentration varied both for formate and NAD and was 0.05–2mM. The assay was always started with the addition of *Rc*FDH (4–8 nM) and the NADH formation was monitored for 60 s. Data obtained were fitted with the Hill function ([y = Vmax × Xn/(Kmn + Xn)], *n* = 1) and Origin software (OriginPro 8.1G SR1; OriginLab Corporation, Northampton, MA, USA).

## Figures and Tables

**Table 1:** Ratios of initial rate constants using unlabeled water versus 50% deuterium oxide (D_2_O) or 50% ^18^O labeled water (H_2_^18^O) demonstrating a kinetic isotope effect for H_2_O/D_2_O.

pH	k_H2O_/k_D2O_	k_H2O_/k_H218O_
6.5	0.92 ± 0.03	0.97 ± 0.026
7	1.21 ± 0.048	1.18 ± 0.037
7.5	1.03 ± 0.033	1.02 ± 0.036
8 (phosphate buffer)	1.69 ± 0.068	1.21 ± 0.059
8 (tris-HCl buffer)	1.45 ± 0.05	1.22 ± 0.054
8.5	1.70 ± 0.062	1.23 ± 0.06
9	1.51 ± 0.048	1.08 ± 0.039
9.5	1.23 ± 0.053	1.10 ± 0.049

**Table 2:** The effect of deuterated solvent and the substrate on the reductive half-reaction of FDH. Rapid reaction kinetic measurements were carried out using a stopped-flow spectrophotometer. A total of 2.5 µM of *Rc*FDH under anaerobic conditions with either D_2_O or H_2_O containing 50 mM phosphate buffer pH/pD 7.9 was used. Formate and deuterated formate (2.5 mM each) were used. Rapid reaction kinetics were obtained by following changes at 445 nm for 2.2 s. k_1_ and k_2_ are the first and second kinetic constants obtained for the reductive half-reaction after a triphasic fit. The slowest third phase was not included as it is independent of formate concentration [15].

	D_2_O				H_2_O		
D_2_O		**k_1_ (s^−1^)**	**k** **_2_ (s^−1^)**	H_2_O		**k** **_1_ (s^−1^)**	**k** **_2_ (s^−1^)**
	DCOO^−^ (2.5 mM)	65.77 ± 1.91	19.44 ± 2.35		DCOO^−^ (2.5 mM)	247.32 ± 18.4	39.44 ± 1.81
	HCOO^−^ (2.5 mM)	91.57 ± 2.41	15.51 ± 1.13		HCOO^−^ (2.5 mM)	263.7 ± 13.88	47.5 ± 2.31

**Table 3:** Kinetic parameters for the steady state kinetics of *R. capsulatus* FDH for formate and monothioformate as substrates (see also Figure 3).

Protein	Substrate	*k*_cat_ (min^−1^)	K_m_ Substrate (mM)	K_m_ NAD^+^ (µM)
*Rc*FDH	Formate	3300 ± 133	0.145 ± 0.025	159.12 ± 12
	Monothioformate	3032 ± 83	3.3 ± 0.24	111.5 ± 24

**Table 4:** Overview of sample composition for the control NMR experiments.

Control Reaction	VEnzyme (µL)	VNAD+ (µL)	VSubstrate (Monothioformate) (µL)	Buffer (pH 9) (µL)
a	0	290	100	210
b	60	0	100	440
c	60	290	0	250
d	0	290	0	310
e	0	0	0	600

a, control reaction without an enzyme (*Rc*FDH); b, control reaction without NAD^+^; c, control reaction without a substrate (monothioformate); c, control reaction without an enzyme (*Rc*FDH) and a substrate (monothioformate); d, control reaction without an enzyme (*Rc*FDH) and substrate; e, control reaction without substrate (monothioformate) and NAD^+^.

## Data Availability

Not applicable.

## Supplementary Materials

The following supporting information can be downloaded at https://www.mdpi.com/article/10.3390/molecules28041537/s1, Figure S1. ^13^C NMR spectrum depicting formate (171.3 ppm), bicarbonate (161.1 ppm), and CO_2_ (125.5 ppm) signals. Figure S2. ^13^C{1H} NMR spectrum confirming the C-H association of formate (170.3, 172.7 ppm). Figure S3. ^13^C NMR example spectrum of the thioformate experiment. Figure S4. ^13^C NMR spectrum of the thioformate reaction mixture after 32 h. Figure S5. ^13^C{1H} NMR spectrum of the thioformate reaction mixture recorded after 32 h. Figure S6. ^13^C NMR spectrm of NaHCO_3_ salt in a buffer at pH 9 at room temperature without enzyme. Figure S7. The control experiment with CO_2_ (unlabeled) dissolved directly into a buffer solution without enzyme and without NAD^+^. Figure S8. Time-dependent series of NMR spectra for the enzymatic conversion of formate. Figure S9. Calibration curve for the 2,3,4,5,6-pentafluoro-benzylbromide(PFBBr)-derivatized formate. Figure S10. Calibration curve for ^13^CO_2_ by using different concentrations of NaH^13^CO_3_ at pH 9.0.

## Sample Availability

Samples of the compounds are available from the authors.
