# Supplementary material for: The Mechanism of Metal-Containing Formate Dehydrogenases Revisited: The Formation of Bicarbonate as Product Intermediate Provides Evidence for an Oxygen Atom Transfer Mechanism
Source: Molecules. 2023 Feb 5;28(4):1537. doi: 10.3390/molecules28041537 (PMC9962302; doi:10.3390/molecules28041537)
Supplement: Supplementary file 1 [file molecules-28-01537-s001.zip › molecules-2122419 - Supplementary Material.pdf]

molecules

Supplimentary information

The mechanism of metal-containing formate dehydrogenases revisited: The formation of bicarbonate as product intermediate provides proof for an oxygen-atom transfer mechanism

Hemant Kumar[§1], Maryam Kosraneh[§2], Siva S. M. Bandaru[2], Carola Schulzke[*2], Silke Leimkühler[*1]

[1] Institute of Biochemistry and Biology, Department of Molecular Enzymology, University of Potsdam, Karl-Liebknecht Strasse 24-25, 14476 Potsdam-Golm, Germany.
[2] Institute of Biochemistry, University of Greifswald, Felix-Hausdorff-Straße 4, 17489 Greifswald, Germany.
[§] These authors contributed equally to this work

[*]**Correspondence should be addressed to C.S. or S.L. (sleimluni@potsdam.de, carola.schulzke@uni-greifswald.de).**

Citation: Kumar, H.; Khosraneh, M.; Bandaru, S.S.M.; Schulzke, C.; Leimkühler, S. The Mechanism of Metal-Containing Formate Dehydrogenases Revisited: The Formation of Bicarbonate as Product Intermediate Provides Evidence for an Oxygen Atom Transfer Mechanism. *Molecules* **2023**, *28*, 1537. https://10.3390/molecules28041537

Academic Editor: Ralf R. Mendel

Received: 10 December 2022
Revised: 31 January 2023
Accepted: 2 February 2023
Published: 5 February 2023

Copyright: © 2023 by the authors. Licensee MDPI, Basel, Switzerland. This article is an open access article distributed under the terms and conditions of the Creative Commons Attribution (CC BY) license (https://creativecommons.org/licenses/by/4.0/).

Supplementary Table and Figure Legends

Supplementary Figure S1: ^{13}C NMR spectrum depicting formate (171.3 ppm), bicarbonate (161.1 ppm) and CO_2 (125.5 ppm) signals**.**

Supplementary Figure S2: $^{13}C\{1H\}$ NMR spectrum confirming the C-H association of formate (170.3, 172.7 ppm).

Supplementary Figure S3: ^{13}C NMR example spectrum of the thioformate experiment.

Supplementary Figure S4: ^{13}C NMR spectrum of the thioformate reaction mixture after 32 h.

Supplementary Figure S5: $^{13}C\{1H\}$ NMR spectrum of the thioformate reaction mixture recorded after 32 h.

Supplementary Figure S6: ^{13}C NMR spectra of $NaHCO_3$ salt in buffer at pH 9 at room temperature without enzyme.

Supplementary Figure S7: Control experiment with CO_2 (unlabelled) dissolved directly into buffer solution without enzyme and without NAD^+.

Supplementary Figure S8: Time dependent series of NMR spectra for the enzymatic conversion of formate.

Supplementary Figure S9: Calibration curve for 2,3,4,5,6-pentafluoro-benzylbromide(PFBBr) derivatized formate.

Supplementary Figure S10: Calibration curve for $^{13}CO_2$ by using different concentrations of $NaH^{13}CO_3$ at pH 9.0.

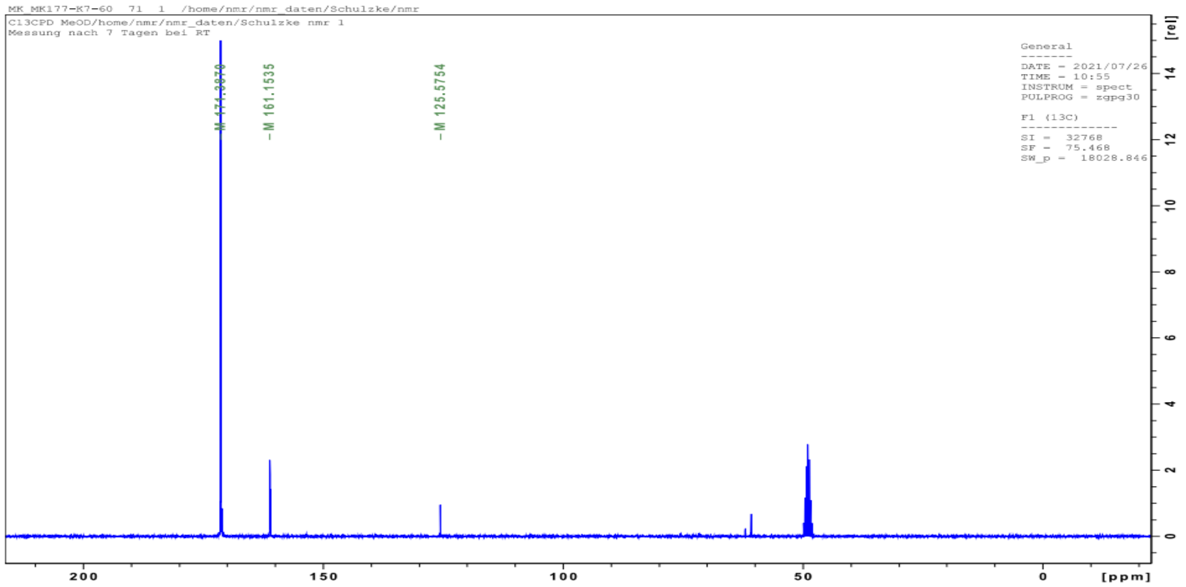

Supplementary Figure S1: ^{13}C NMR spectrum depicting formate (171.3 ppm), bicarbonate (161.1 ppm) and CO_2 (125.5 ppm) signals in water and referenced against MeOD (quasi-internal yet separated standard in an insert tube).

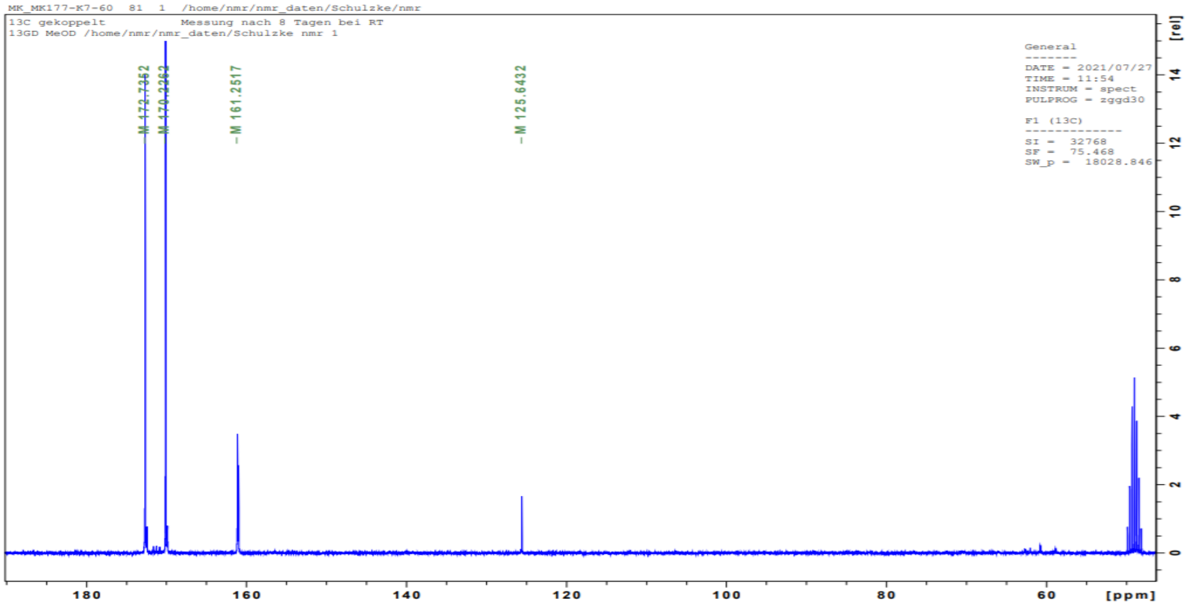

Supplementary Figure S2: **$^{13}C\{1H\}$ NMR spectrum confirming the C-H association of formate** (170.3, 172.7 ppm) and absence of C-H bonds in bicarbonate (161.2 ppm) and CO_2 (125.6 ppm) in water and referenced against MeOD (quasi-internal yet separated standard in an insert tube).

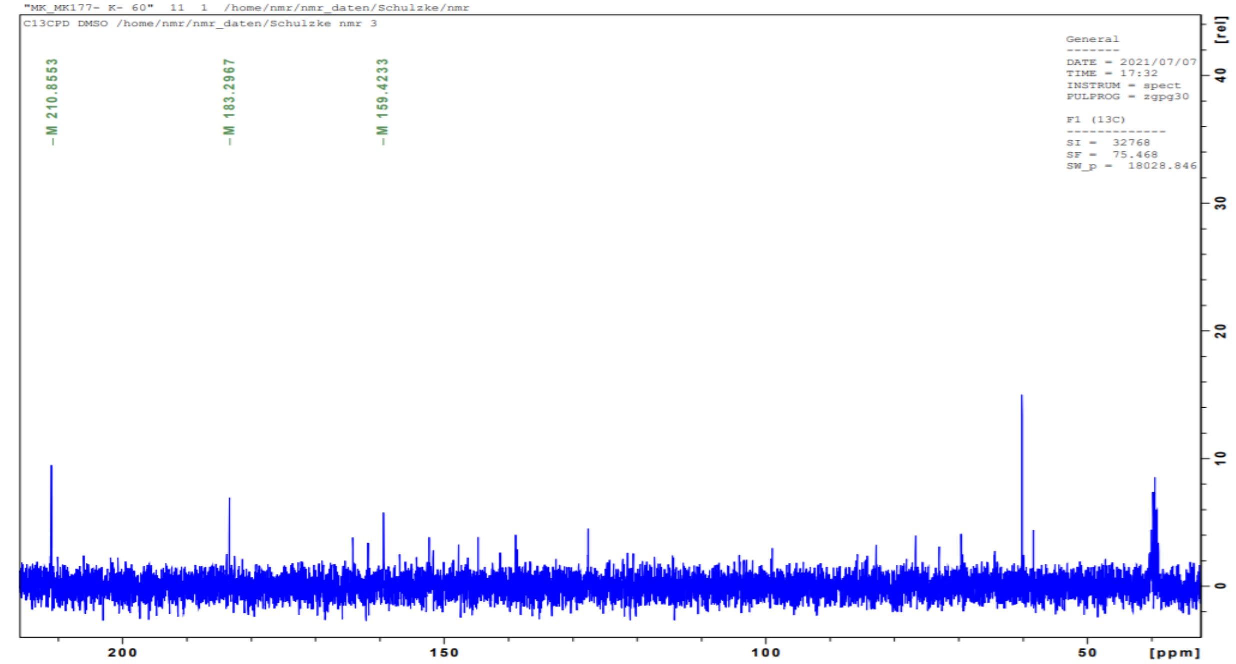

Supplementary Figure S3: **^{13}C NMR example spectrum of the thioformate experiment.** The NMR signals at 211, 183, and 159 ppm confirm the concomitant presence of thioformate, thiocarbonate and COS, respectively, in a reaction mixture in water and referenced against DMSO (quasi-internal yet separated standard in an insert tube). Unassigned resonances go back to buffer components and NAD^+/NADH.

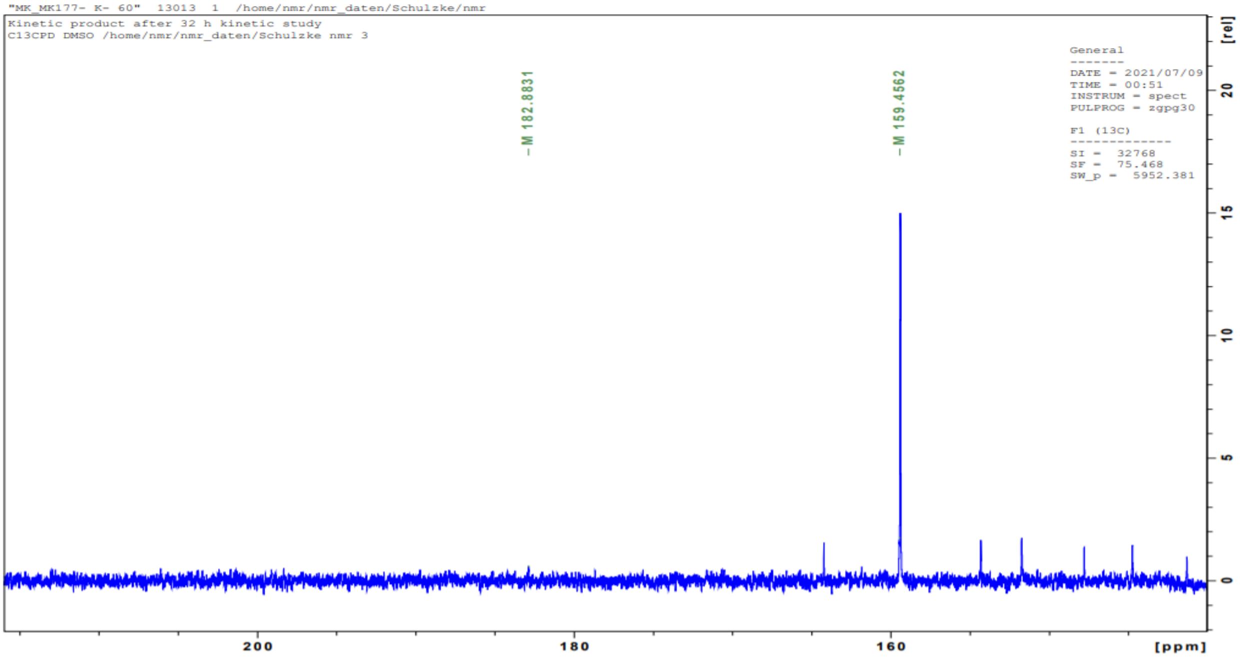

Supplementary Figure S4: **^{13}C NMR spectrum of the thioformate reaction mixture after 32 h.** The thioformate starting material is completely consumed (absence of peak at 211 ppm), mild quantities of thiocarbonate intermediate at 183 ppm and a higher abundance of COS (peak at 159 ppm) were observed.

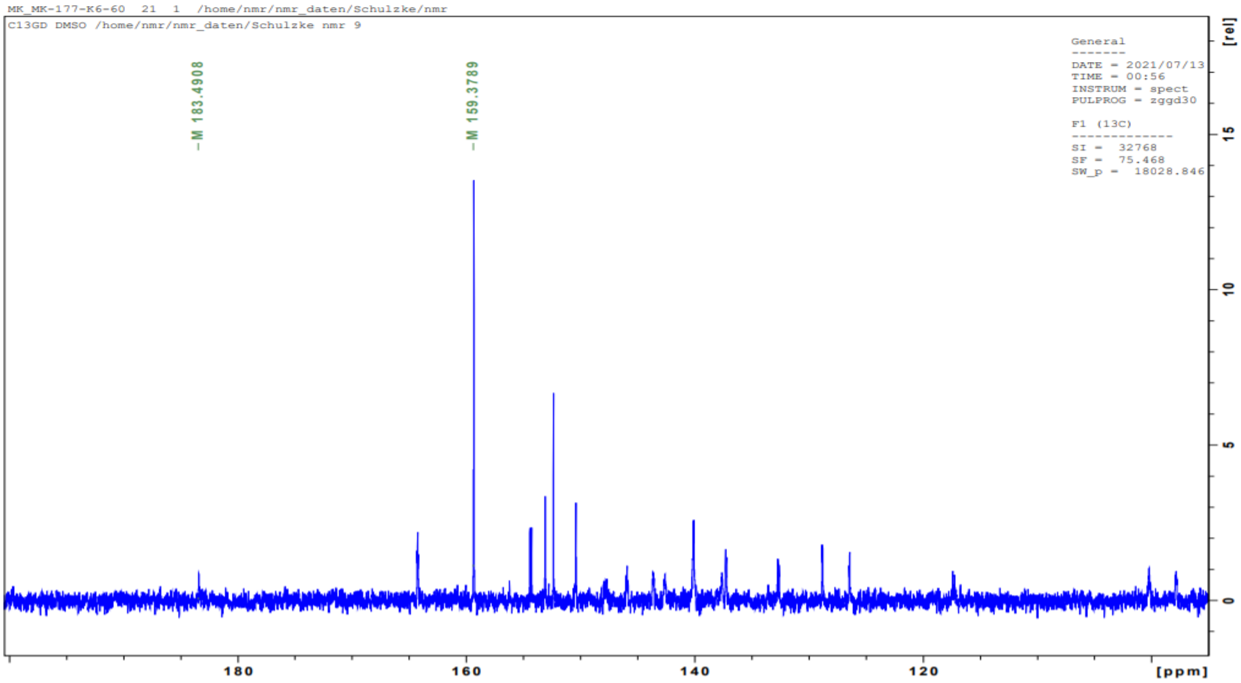

Supplementary Figure S5: **^{13}C{1H} NMR spectrum of the thioformate reaction mixture recorded after 32 h.** The peaks at 183 ppm (thiocarbonate) and 159 ppm (COS) are not involved in any C-H coupling, which further confirms the absence of C-H bonds in these species.

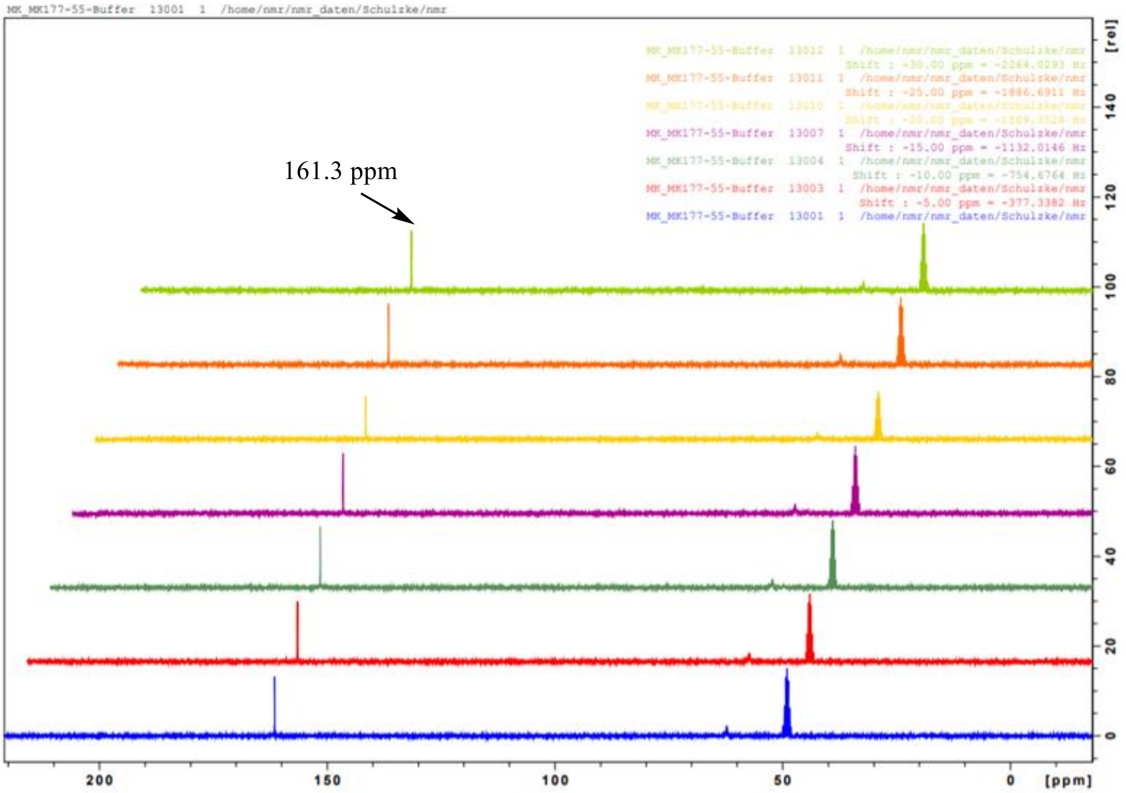

Supplementary Figure S6: **^{13}C NMR spectra of $NaHCO_3$ salt in buffer at pH 9 at room temperature without enzyme.** The spectra were recorder at regular intervals of 1 h for 15 h with MeOD as internal standard. A resonance signal **at 161.3** ppm represents bicarbonate; generation of CO_2 was not observed. In this case unlabelled HCO_3^- was used and it is likely that the equilibrium concentration of CO_2, which is very low as shown in the time dependent experiments does not result in a signal rising above the noise considering a natural abundance of ^{13}C of only 1.1%.

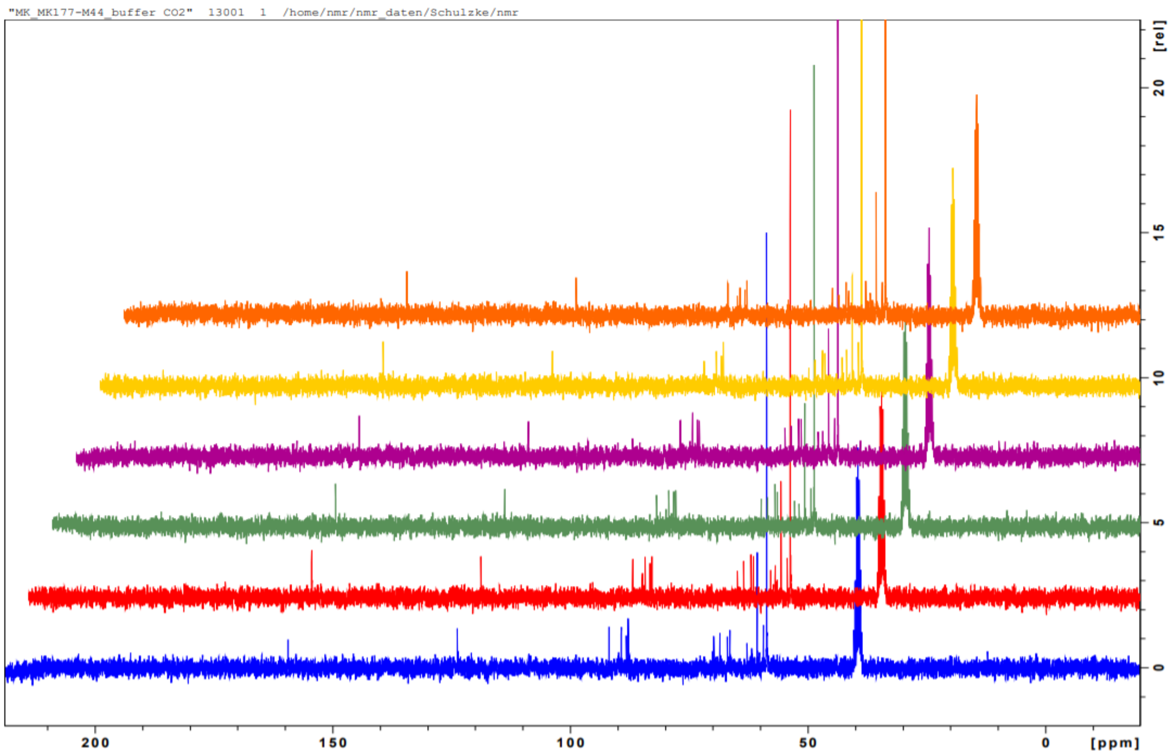

Supplementary Figure S7: Control experiment with CO_2 (unlabelled) dissolved directly into buffer solution without enzyme and without NAD^+. The formation of bicarbonate is already evident in the first spectrum. An apparent equilibrium between CO_2 and HCO_3^- with a slight excess of bicarbonate over CO_2 is reached relatively quickly. However, the bicarbonate resonance at 161 ppm is initially decidedly smaller than that of CO_2 at 124 ppm and then increases to a ratio which is still much closer to unity compared to that observed in the enzymatic experiment. In the enzyme experiments, the ratio FELL until reaching the equilibrium with a decidedly higher abundance of bicarbonate relative to CO_2. In addition, the control experiment was carried out at 25 °C while the enzymatic formate oxidation was studied at 5 °C. The bicarbonate/CO_2 equilibration is temperature dependent and much faster at temperatures around room temperature than in the cold. These observations further imply that the presence of enzyme and NAD^+/NADH affects this equilibrium, which is likely also maintained by enzyme activity in either direction. The ^{13}C-spectra of this control experiment were measured in regular intervals of 2 h with a pause time of 3 minutes and referenced against DMSO as quasi-internal yet separated standard in an insert tube.

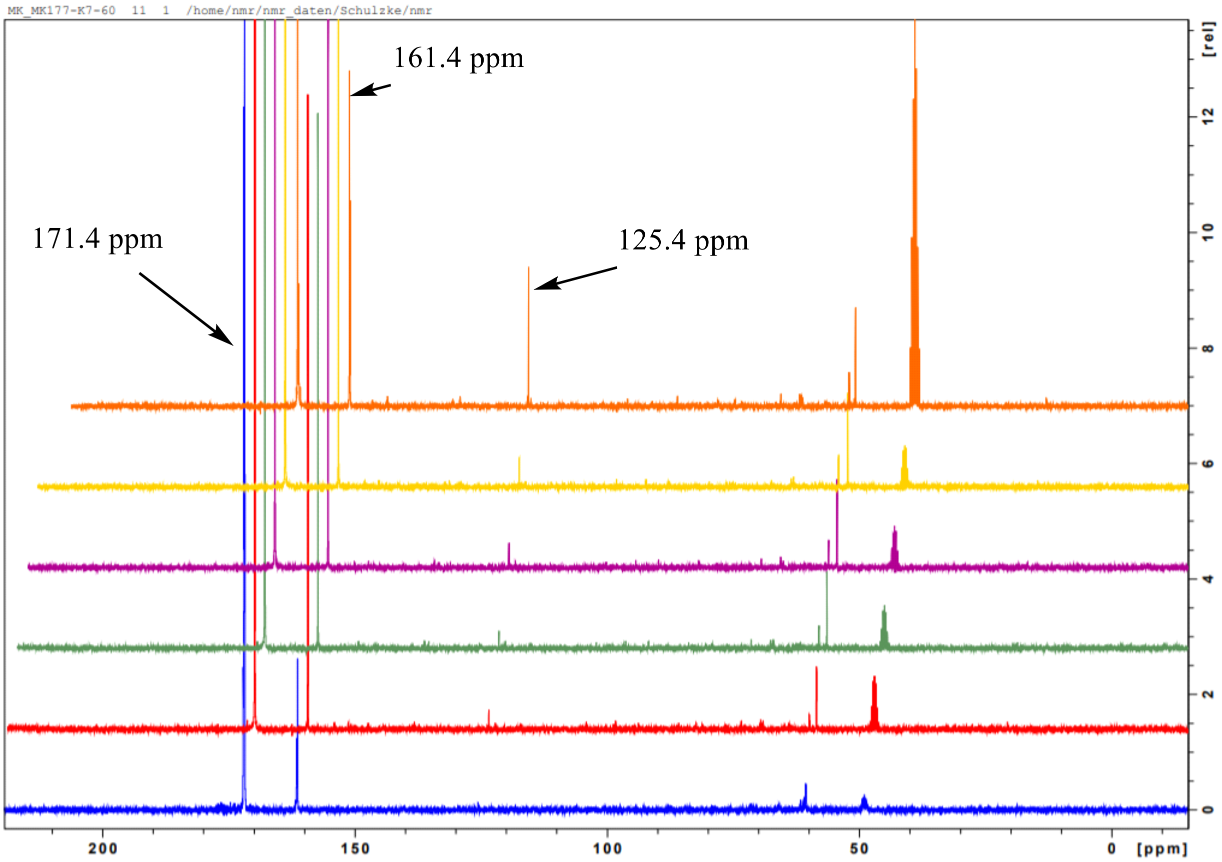

Supplementary Figure S8: Time dependent series of NMR spectra for the enzymatic conversion of formate. Reaction monitoring was carried out with ^{13}C NMR measurements of a short duration of 25 minutes per spectrum, with azide inhibited enzyme, at pH 9 and at 5 °C. The last spectrum in the series was recorded as coupled measurement to confirm the absence of C-H bond for initial product bicarbonate and second product CO_2.

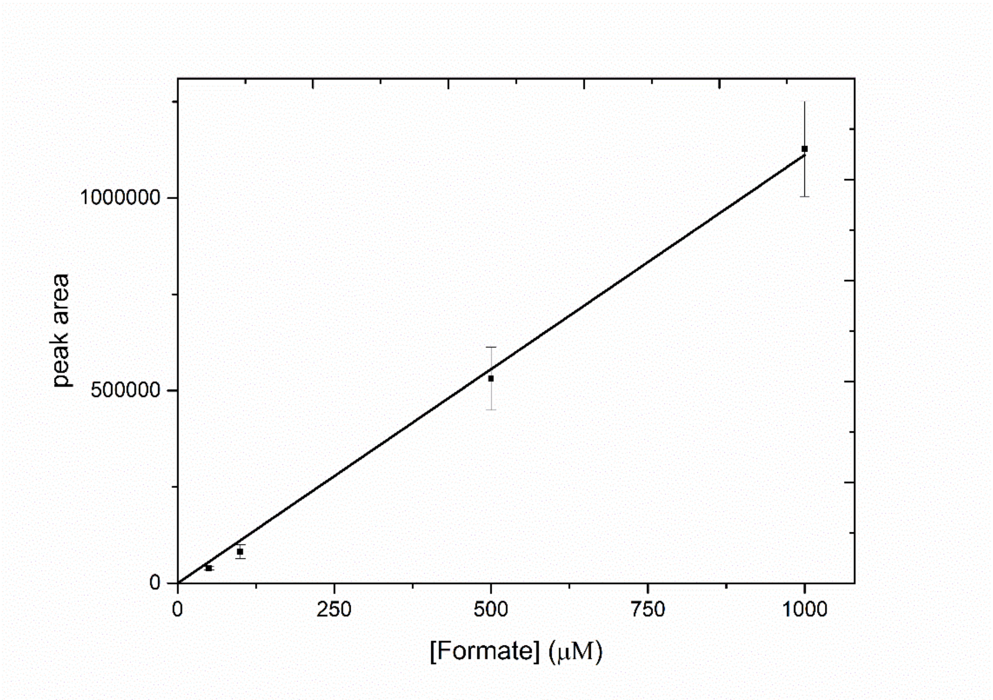

Supplementary Figure S9: Calibration curve for 2,3,4,5,6-pentafluoro-benzylbromide(PFBBr) derivatized formate. 100 μL of different concentrations of formate solution were mixed with 50 μL of 325 mM phosphate buffer, pH 8.5. Then 365 μL of 100 mM PFBBr (prepared in acetone) was added. This solution was vortexed for 1 min followed by heating at 60 °C for 20 min. After cooling down to room temperature, 500 μL of n-hexane was added and vortexed for 1 min. Phases were separated by centrifuging at 13000 rpm for 1 min and the upper organic phase was carefully pipetted into 2mL insert containing GC vials. The samples were analysed by using GC-MS QP2010 SE (Schimadzu). Sample volumes of 1 μL were used in the DB-WAX UI column (30m × 0.32mm × 0.25μm, Agilent). Selected ion monitoring (SIM) mode for detection of m/z = 226 was used for MS analysis.

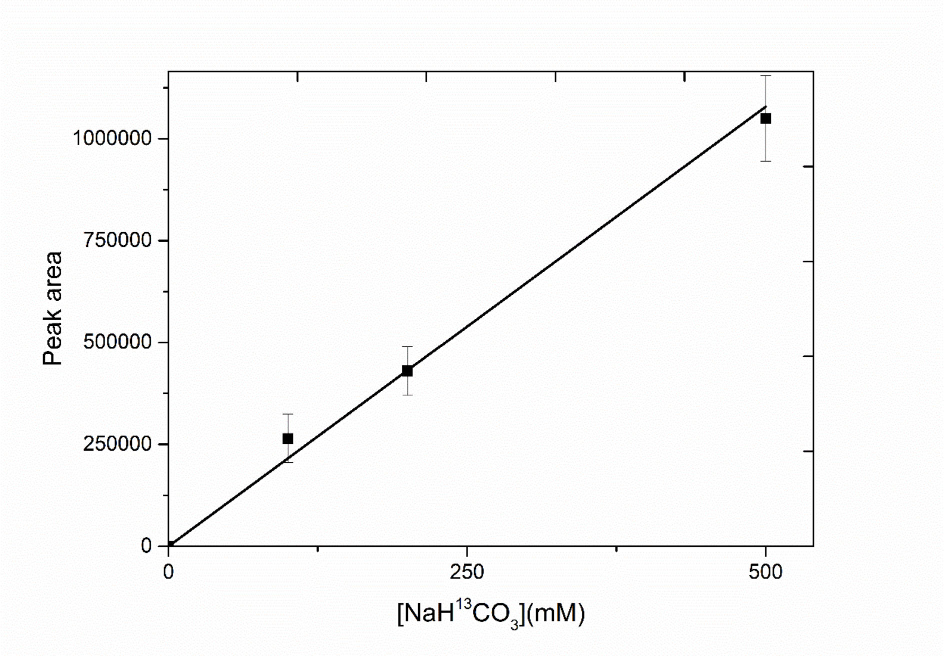

Supplementary Figure S10: **Calibration curve for $^{13}CO_2$ by using different concentrations of $NaH^{13}CO_3$ at pH 9.0.** 100 μL of different $NaH^{13}CO_3$ standard concentrations were prepared in 2mL GC vials by using 1M $NaH^{13}CO_3$ solution in 100 mM Tris-HCl. 1-5 μL of headspace samples were analysed by using GC-MS QP2010 SE (Schimadzu) modified for headspace samples. DB-WAX UI

column was used at a temperature of 30 °C. MS analysis method detected the m/z = 44 and 45 for CO_2 and $^{13}CO_2$ respectively.
